# Matched-Compliance Benchmarking of Perforated and Uniformly Thinned Substrates for Piezoelectric Cantilever Energy Harvesters

**DOI:** 10.3390/mi17101158

**Published:** 2026-09-30

**Authors:** Saad F. Almokmesh, Bashar B. Alzuwayer

**Affiliations:** 1 Department of Mechanical Power and Refrigeration Technology, College of Technological Studies, Public Authority for Applied Education and Training (PAAET), Kuwait City 70654, Kuwait; 2 Department of Automotive Mechanics and Marine Engineering Technology, College of Technological Studies, Public Authority for Applied Education and Training (PAAET), Kuwait City 70654, Kuwait

**Keywords:** piezoelectric energy harvesting, cellular substrate, perforated beam, matched compliance, distributed-parameter model, design optimization, self-powered sensors, wireless sensor networks

## Abstract

Cellular and perforated substrate roots are stated to improve the output of piezoelectric cantilever energy harvesters, which serve as the primary vibration power source for self-powered wireless sensor nodes. The present work applies stricter control: a uniformly thinned root of equal substrate compliance. A single geometric family, defined by hole–footprint fractions and depth, contains through-cuts, skin-closed pockets and the thinned band. The family is analyzed with closed-form section mechanics, a validated segmented distributed-parameter model with active-coverage bounds for electrodes bridging holes, and coupled-field finite element analysis, and it is optimized in footprint, depth, thickness profile, and load in matched and frequency-free frames. At matched compliance and equal assumed damping, 1 m/s^2^ and 1 MΩ, the band delivers 27.8 µW against 20.7 (through-cut) and 25.8 µW (pocket), a 62% gain over the solid reference at equal footprint, at the lowest peak substrate stress of the three, and optimization terminates at the no-hole boundary. The through-cut’s +20% gain over the solid baseline reproduces the literature and is traced to root compliance. Sensitivity studies bound the claim. The power ordering holds for all patch thicknesses and coverages in the weak-coupling regime and at equal modal damping, reversing only if the band’s damping exceeds that of the pocket’s thinner skin and slender ribs. It flips in favor of the pocket under strong coupling at optimal load. The stress ordering reverses only with a damping contrast of 24–62%, an order of magnitude larger. Coupled finite element checks at three optimized designs confirm the model-frame conclusions: the best real-hole design at matched compliance delivers 16% less power than the plain band, while for a front-loaded thickness profile, a thinned root still delivers 9% more at 1.6-times the peak stress. Within this envelope, the benefits belong to thinning, not preformation.

## 1. Introduction

The cantilevered piezoelectric harvester, which is made up of a clamped beam, a bonded ceramic, and a resistive load, is still the standard way to use ambient vibration as a source of energy. Roundy and Wright demonstrated two decades prior that a centimeter-scale bender can power wireless electronics using machine vibrations [1]; Beeby et al. articulated the inertial-generator challenge that all such devices address [2], and the distributed-parameter theory proposed by Erturk and Inman [3,4] provided the operational framework for the field. The majority of performance improvements have arisen from geometry rather than materials, leaving a large literature on which geometry has contributed, which we group into five strands.

The oldest lever is the planform. Baker, Roundy and Wright showed that a trapezoidal beam equalizes the strain distribution and yields roughly 30% more power per unit volume than a rectangular one [5]. Shape-optimized bimorphs reinforced the idea [6]; topology optimization added stress limits to the search [7]; the literature on beam shapes is now large enough to warrant its own reviews [8].

The more active branch designs the substrate itself. Li, Kuang, and Zhu initiated the study by forecasting a 2.76-fold increase in power from an auxetic core, attributing the negative Poisson’s ratio that engages both transverse and longitudinal modes [9], while Ferguson et al. provided empirical validation [10]. The family has expanded into auxetic boosters [11], porous auxetic inserts [12], hexachiral [13] and re-entrant honeycomb substrates [14], perforated-chirality hybrids [15], three-dimensional metastructures [16], graded cells [17], and rotating-triangle lattices [18]; this branch is thoroughly documented [19]. Reported increases range from a few percent to over an order of magnitude, consistently accompanied by a beneficial reduction in resonance.

A concurrent thread attains comparable figures with simpler geometry. Basic perforations enhance output [20]; a solitary rectangular aperture in the substrate diminishes the eigenfrequency while increasing voltage and power in both theoretical and experimental settings [21]; hollow structures [22], multi-orifice configurations [23], and slitted beams [24] exhibit a similar trend, as does our research on slitted cantilevers and their optimization [25,26]. The literature on effective properties provides the interpretation that perforated segments possess classical equivalent constants [27,28], cellular harvesting bands can be homogenized similarly [29], and recent research linking their advantages to fundamental principles consistently points to effective stiffness rather than cell morphology [30,31,32]. Diminishing the rigidity of the substrate under the electrode focuses the curvature at the transducer’s location; the cells represent one method of achieving this softening.

If such interpretation is accurate, an earlier body of work gains immediate significance: a softening via density. Segmented sections alter and expand the reaction [33], substrate-thickness investigations chart the output-versus-durability balance [34], tapers have been designed for consistent stress [35], variable-thickness composite beams are developed [36], and tapered-auxetic hybrids are present [37]. Thinning is the most economical form of compliance (a single milling pass, no stress concentrators and a continuous bonding surface), yet it has never served as the benchmark for evaluating architected roots.

The criteria for comparing harvesters remain ambiguous. Normalized power density serves as the conventional cross-device measure [38]; there have been calls for uniform reporting [39]; closed-form analyses delineate the limits of possible power density based on permissible stress [40]; and the fatigue of substrate and ceramic is acknowledged as a limiting factor [41,42]. Each of these issues indicates a common direction: a comparison that overlooks stress or selects a weak baseline tends to favor the treated design. None of it has been directed at the claims made for the architected substrate itself.

This study addresses this gap. The literature on cellular substrates lacks a matched-compliance control; the closest analogous examples are the stiffness-matching discussions for solid beams [30,32] and limited sandwich evaluations [43]. Matched-frequency benchmarking has a well-established foundation: Patel and colleagues maintained the natural frequency constant explicitly to facilitate impartial comparisons among various harvester designs in their coverage studies [44] and subsequently developed equal-frequency devices by modifying the substrate thickness [45]; the current protocol applies that rigor to cellular roots by aligning substrate compliance instead. We, therefore, perform the missing control. A single geometric family (rectangular features of footprint *f_x_ p_x_* × *f_z_ p_z_* and depth *βh_s_* at the root of a unimorph) contains through-thickness perforations, blind cavities sealed by a skin and, in the absence of holes, a uniformly thinned strip. All designs are calibrated to the identical substrate compliance and evaluated as complete harvesters using three-dimensional coupled-field finite elements alongside a validated segmented electromechanical model; this evaluation is subsequently generalized from three designs to the entire family through direct optimization of the footprint, depth, thickness profile, and load, in both the matched and conventional frequency-free frameworks. Along the way, we provide a benchmarking protocol for reuse, closed-form mechanics for the stress and stiffness of each topology, an active-coverage analysis featuring two bonding limits for electrodes that span holes, and a design principle corroborated by the results, as delineated in Section 5.8. With constant compliance and a fixed transducer, root geometry serves as a stress and tuning mechanism, rather than a power mechanism. Section 2 elaborates on the analytical framework, Section 3 addresses the finite element benchmark, Section 4 discusses the validation process, and Section 5 presents the findings.

Every one of these gains is measured against a solid beam. That baseline conflates two things a root treatment does once: it adds compliance, which any thinner root would also add, and it redistributes strain, which is the effect specific to the pattern. The field, therefore, has no measurement of what a hole pattern contributes beyond the compliance it costs and, hence, no basis for deciding whether the pattern is worth cutting. This study supplies that control. Every performed root is compared against a uniformly thinned root of identical substrate compliance, so that the residual difference is attributable to topology alone. The same devices are then reported in the frames in which harvesters are conventionally compared: matched device frequency, optimal load, mass-normalized power and normalized power density, so the attribution and the design value are read together.

The application frames the numbers. A duty-cycle wireless sensor node operates at the microwatt level during sleep and consumes milliwatts only intermittently. So, a centimeter-scale root-treated cantilever producing tens of microwatts at a 1 m/s^2^ machine vibration intensity is a node that requires no battery replacement. The best root treatment identified here raises the budget by 62% over a solid beam of the same footprint at equal modal damping while lowering the peak substrate stress, power and durability moving together rather than trading off. Which root treatment extracts the most from a given footprint is, therefore, a power-supply question for self-powered sensing, not only a mechanical one, and this work answers it under controlled comparison.

## 2. Analytical Model

### 2.1. A Single Geometry Family

**Figure 1:**
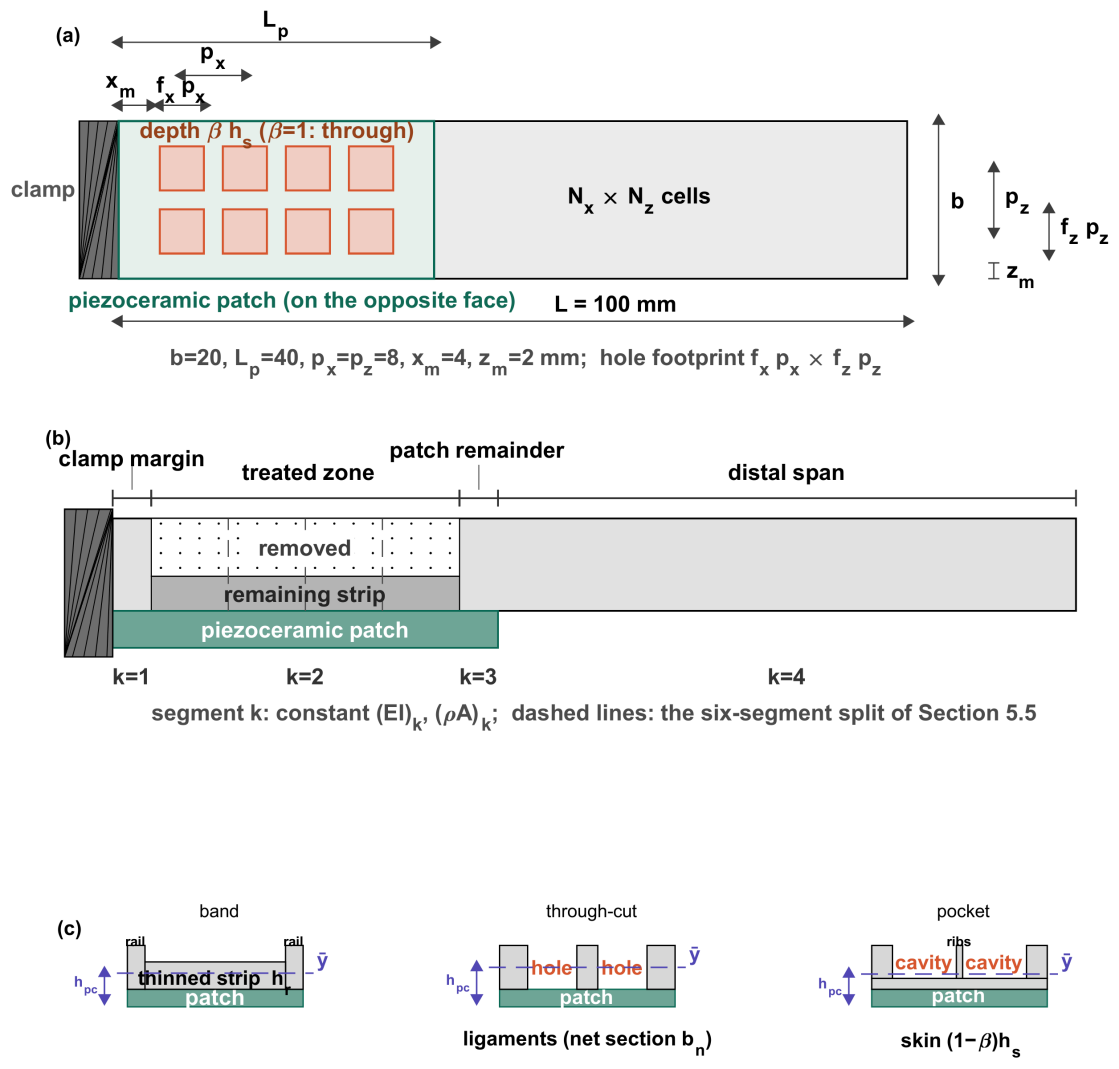
The harvester family and vocabulary. (**a**) Plan view and parameterization: piezoceramic patch of length L_p_ on the face opposite the features, N_x_ × N_z_ cells of pitch p_x_, p_z_ with margins x_m_, z_m_, hole footprint f_x_p_x_ × f_z_p_z_ and depth βh_s_ (β = 1: through). (**b**) Side view: the four segments of the model, clamp margin, treated zone, patch remainder and distal span, each of constant (*EI*)k and (*ρ*A)k; dashed lines mark the six-segment split of Section 5.5. (**c**) Cross-sections of the treated zone at a feature station: the band’s thinned strip between full-height rails, the through-cut’s ligaments forming the net section b_n_, and the pocket’s skin, ribs cavities; ȳ is the neutral axis and h_pc_ the coupling arm to the patch midplane.

Figure 1 illustrates the device and its parameterization. A steel-like substrate (*E_s_* = 100 GPa, *ν* = 0.3, *ρ_s_* = 7165 kg/m^3^) of length *L* = 100 mm, width *b* = 20 mm, and thickness *h_s_* = 1.0 mm is clamped at x = 0 and carries a PZT-5A2 patch. The substrate constants and the modal damping ratio ζ = 0.02 are derived from the reference device of our previous slit-ted-cantilever investigations [25,26] instead of being associated with a specific alloy; the impact of ζ on each principal margin is detailed in Section 5.6. The patch exhibits a thickness of *h_p_* = 0.4 mm across the root for the range 0 ≤ *x* ≤ *L_p_* = 40 mm. In the designated area *x_m_ ≤ x ≤ L_p_* − *x_m_* (*x_m_* = 4 mm), a *N_x_* × *N_z_* = 4 × 2 array of rectangular elements with pitches *p_x_* = *p_z_* = 8 mm, bordered by edge margins *z_m_* = 2 mm, is excised from the surface opposing the patch. Every characteristic has an in-plane footprint *f_x_ p_x_* × *f_z_ p_z_* and a depth *β h_s_*. A single parameterization encompassing all situations is what makes the comparison controlled: any response difference is traceable to topology, not accidental geometry.

Throughout, the vocabulary follows Figure 1. Along the beam, the device has four segments: the solid clamp margin, the treated zone carrying the feature array, the solid span (Figure 1b). Within the treated zone, the cross-section differs by topology (Figure 1c): the uniformly thinned band consists of a thinned strip between two full-height edge rails. The through-cut carries the bending moment through the ligaments between holes, whose combined width is the net section b_n_. The pocket is closed on the bonding face by a continuous skin of thickness *(*1 − *β)h_s_,* leaving ribs between cavities. For a station cross-section at a given x, a feature station crosses a hole or cavity, with a solid station being the material between them. The configuration carries no tip mass, so that the root treatment is the only difference between devices; the segmented model of Section 2.3 accepts a tip mass boundary condition, and Section 5.6 reports the tip mass case.

The uniformly thinned band is not a flat segment: the edge margins *z_m_* maintain their full height and function as rigid supports beside the thinned strip. The neutral axis and stiffness are derived from the composite equations, using *A_r_* = 2*z_m_h_s_* (rails, centroid at *h_s_*/2) and *A_c_* = (*b* − 2*z_m_*)*h_r_* (strip, centroid at β *h_s_* + *h_r_*/2, measured from the thinned face).

(1) $$\bar{y}=\frac{A_{r}\;h_{s}/2+A_{c}\;\left(\beta h_{s}+h_{r}/2\right)}{A_{r}+A_{c}},\;\;{\left(\mathrm{EI}\right)}_{r}^{\mathrm{band}}=E_{s}\left[\frac{2z_{m}h_{s}^{3}}{12}+A_{r}{\left(\frac{h_{s}}{2}-\bar{y}\right)}^{2}+\frac{\left(b-2z_{m}\right)h_{r}^{3}}{12}+A_{c}{\left(\beta h_{s}+\frac{h_{r}}{2}-\bar{y}\right)}^{2}\right],$$

And the surface stress of the thinned strip follows the plate form

(2) $$\sigma^{\mathrm{band}}=\frac{6M}{bh_{r}^{2}}=\frac{\sigma_{0}}{{\left(1-\beta \right)}^{2}},\;\;{\left(\mathrm{EI}\right)}^{\mathrm{plate}}=\frac{E_{s}bh_{r}^{3}}{12}\;\leq \;{\left(\mathrm{EI}\right)}_{r}^{\mathrm{band}},$$

The plate rigidity is preserved in the code just as a designated lower limit. The rails are significant in quantitative terms: at the corresponding depth *β* = 0.3667 (*h_r_* = 0.633 mm), Equation (1) yields $\bar{y}$ = 0.632 mm and *I* = 0.768 mm^4^, compared to the bare plate’s 0.423 mm^4^, resulting in a factor of 1.82; the solid beam’s *bhs*^3^/12 = 1.667 mm^4^ establishes the benchmark. All correspondences in this document use Equation (1). At a station crossing a through-hole array, the load-bearing width reduced to *b_n_* = *b* − *N_z_ f_z_ p_z_*. Under a shared moment *M*, the total rotation accrued over a single pitch is the aggregate of the contributions from both stations.

(3) $$\Delta \vartheta =\frac{M\left(1-f_{x}\right)p_{x}}{{\left(\mathrm{EI}\right)}_{\mathrm{full}}}+\frac{Mf_{x}p_{x}}{{\left(\mathrm{EI}\right)}_{\mathrm{net}}}\equiv \frac{Mp_{x}}{{\left(\mathrm{EI}\right)}_{r}^{\mathrm{thr}}},$$

Compliance, not stiffnesses, average, so the net station dominates. The mass carries no such series structure and averages arithmetically

(4) $$A_{\mathrm{mass}}=bh_{s}\left[\left(1-f_{x}\right)+f_{x}\;\frac{b_{n}}{b}\right].$$

The same net section gives the net section lower band

(5) $$\sigma^{\mathrm{thr}}\;\geq \;\frac{b}{b_{n}}\;\sigma_{0},\;\;{\left(\mathrm{EI}\right)}_{r}^{\mathrm{thr}}=\frac{E_{s}bh_{s}^{3}}{12}{\left[\left(1-f_{x}\right)+\frac{f_{x}}{\;b_{n}/b\;}\right]}^{-1},$$

The stiffness follows from Equation (3). In matched design *b_n_* = 8.8 mm, *b*/*b_n_* = 2.27, the bracket evaluates 0.529 (so *I_eff_* = 0.881 mm^4^), while Equation (4) gives *A_mass_* = 0.608 *bh_s_*. That asymmetry, harmonic in stiffness, arithmetic in mass, is why perforation buys compliance so much faster than it sheds mass. The blind-pocket station is composed of three parts, each entering with its own area and centroid (heights measured from the pocketed face, the bonding face is at *y = h_s_*).

(6) $$\begin{array}{lllll} \text{margins}: & A_{m} & =2z_{m}h_{s}, & {\bar{y}}_{m} & =h_{s}/2,\\ \text{skin}: & A_{s} & =\left(b-2z_{m}\right)\left(1-\beta \right)h_{s}, & {\bar{y}}_{s} & =\beta h_{s}+\left(1-\beta \right)h_{s}/2,\\ \text{ribs}: & A_{\mathrm{rib}} & =\left(1-f_{z}\right)\left(b-2z_{m}\right)\beta h_{s}, & {\bar{y}}_{\mathrm{rib}} & =\beta h_{s}/2, \end{array}$$

which, at the matched design (*f* = 0.909, β = 0.75), reaches 4.0 and 1.09, respectively. Its neutral axis $\bar{y}$ and second moment *I_w_* then follow from standard composite formulae

(7) $$\bar{y}=\frac{\sum A_{i}{\bar{y}}_{i}}{\sum A_{i}},I_{w}=\sum \left(I_{i}+A_{i}{\left({\bar{y}}_{i}-\bar{y}\right)}^{2}\right).$$

The results below are driven by two features of Equation (7). The stiff skin is on the far face, so $\bar{y}$ moves toward it: the lower side of the rib has a larger fiber distance on the reduced section, and the nearer face, seen by the piezoceramic, has a smaller one. For the matched design, Equation (7) yields $\bar{y}$ = 0.650 mm and *I_w_* = 0.780 mm^4^. The closed-skin root, therefore, pays twice, more stress in the critical fiber, less strain in the transducer, and the second penalty is quantified below via the lever arm *h_pc_*.

### 2.2. Reduced Piezoelectric Constants

The PZT-5A2 data (elastic *c^E^*, piezoelectric e, permittivity *ε^S^*; poling through the thick-ness) are reduced to the one-dimensional beam state (*σ_x_* the only stress, field *E_y_* only) by numerically eliminating the transverse strains from the full constitutive matrices, giving

(8) $${\bar{E}}_{p}=61.0\;\mathrm{GPa},\;{\bar{e}}_{31}=-10.43\;C\;m^{-2},\;{\bar{\varepsilon }}^{S}=1498\;\varepsilon_{0}.$$

The condensation lands exactly on the accepted uniaxial PZT-5A values [3], giving a free consistency check between the finite element material input and the beam model.

### 2.3. Segmented Electromechanical Model and Active Coverage

Each device is a stepped Euler–Bernoulli beam, a chain of *n* uniform segments with constant properties: solid clamp margin, treated zone with the properties in Section 2.2, solid patch remainder, and bare distal span (*n* = 4, Figure 1b; the thickness-profile study of Section 5.5 splits the treated zone into four cells, *n* = 6). On segment *k*, with constant (*EI*)*_k_* and (*ρA*)*_k_*, free vibration at frequency *ω* abides

(9) $${\left(\left(\mathrm{EI}\right)\right)}_{k}\;\frac{d^{4}\phi }{d\xi^{4}}=\omega^{2}{\left(\mathrm{\rho A}\right)}_{k}\;\phi ,\;\;\lambda_{k}={\left[\frac{\omega^{2}{\left(\mathrm{\rho A}\right)}_{k}}{{\left(\left(\mathrm{EI}\right)\right)}_{k}}\right]}^{1/4},$$

whose general solution on the segment is the standard four-term combination trigonometric and hyperbolic function,

***ϕ_k_***(***ξ***) = ***C***_1***k***_**cos**(***λ_k_ξ***) + ***C***_2***k***_**sin**(***λ_k_ξ***) + ***C***_3***k***_**cosh**(***λ_k_ξ***) + ***C***_4***k***_**sinh**(***λ_k_ξ***), (10)

Each segment contributes four constants, giving 4n unknowns in total for the mode shape (16 here). These are fixed by the clamp (φ = φ′ = 0), the free tip (φ″ = φ‴ = 0) and, at every interface, continuity of deflection, slope, moment (*EI*)φ″ and shear (*EI*)φ‴, the section-weighted pair being what jumps a stepped beam cannot inherit from a uniform one. The modal equations provide the coupled response. The characteristic determinant is of size 4n × 4n, and its roots yield the natural frequencies

(11) $${\ddot{\eta }}_{r}+2\zeta_{r}\omega_{r}{\dot{\eta }}_{r}+\omega_{r}^{2}\eta_{r}-\theta_{r}v=f_{r}\left(t\right),C_{p}\dot{v}+\frac{v}{R}+\sum_{r}\theta_{r}{\dot{\eta }}_{r}=0,$$

**Figure 2:**
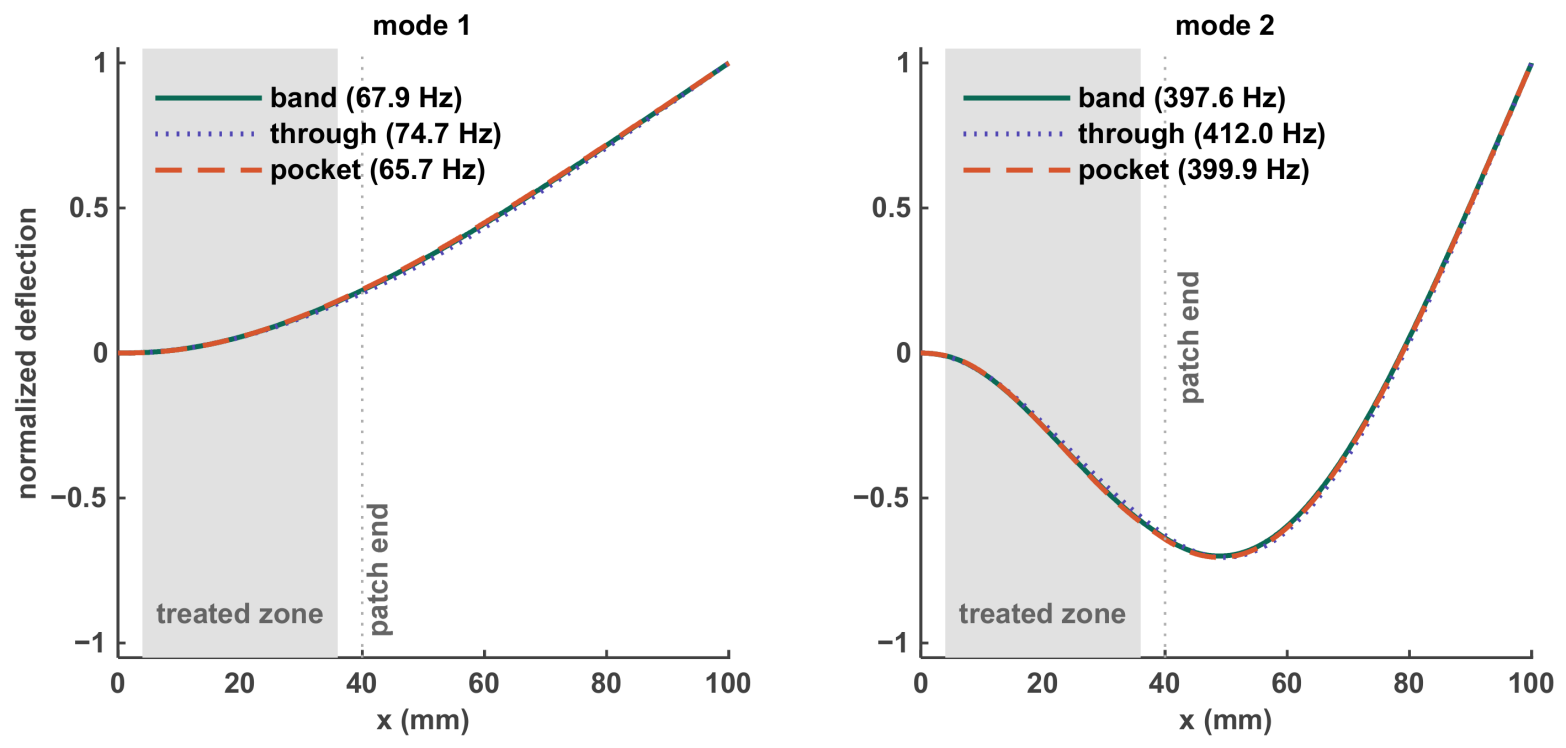
Computed mode shapes of the three devices (segmented model, mass-normalized, tip-positive). Mode 1 concentrates curvature in the treated zone under the electrode, the harvesting mechanism, while mode 2 lies a factor of ∼5.8 higher for every design.

Here, *η_r_* is the coordinate of the mass-normalized mode (mass normalized so that ∫0LρA φr2 dx=1, three modes retained; the first two computed mode shapes of the three devices are shown in Figure 2). The base excitation of amplitude a forces each mode through −a∫0LρA φr2 dx=−aΓr. The patch capacitance is *C_p_* = ε*^S^bL_p_*/*h_p_* = 26.5 nF. The modal coupling is assembled segment wise,

(12) $$\theta_{r}=-{\bar{e}}_{31}\;b\sum_{k}\alpha_{k}\;h_{\mathrm{pc},k}[{\phi_{r}}^{\prime }\left(x\right){]}_{x_{k}}^{x_{k+1}}.$$

Here, *h_pc_***_,_***_k_* is the neutral-axis-to-patch-midplane distance of segment *k*, the coupling’s lever arm, and it ranks the topologies before any dynamics is solved: with the patch attached, *h_pc_* is 0.563 mm for the solid section, 0.479 (through-cut), 0.423 (band) and 0.358 mm for the pocket, whose stiff far-face skin drags the neutral axis furthest from the transducer, a 36% loss of arm against the solid section. Here, *αk* is its active coverage, the fraction of electrode bonded to substrate. For the band and for the closed-skin pocket, *α* = 1 by construction. For the through-perforated root, the bonded fraction is geometric, and two coverage numbers are relevant to the device studied: the bonded fraction of the whole electrode, 1 − *N_x_N_z_ f_x_ f_z_*,*p_x_p_z_*/(*L_p_ b*) = 0.686 (independently corroborated by the finite element bonding count: the node-by-node interface constraint equations of Section 3 tie the through-cut patch at 16,863 of the full-face 23,220, a bonded fraction of 0.73), and the coverage of the treated segment alone, α_2_ = 1 − *N_x_N_z_ f_x_ f_z_*,*p_x_p_z_*/[(*L_p_* − 2*x_m_*)*b*] = 0.608, which is the value used in Equation (12) and the rigid-α bound. However, the electrode spanning each hole is not necessarily inert: it is still connected to the bonded regions and can transmit membrane strain. The truth is bracketed between two limiting idealizations, the classical rigid*-α* model (bridging patch inactive) and a force-continuity bound (α = 1, bridging patch fully strained). Section 4 shows that, once the common model-to-finite-element offset is removed, the finite element device lies inside this bracket, near the force-continuity bound.

Under harmonic excitation at frequency ω, the modal equations reduce, with *D_r_* = ω*_r_*^2^ − ω^2^ + 2*j*ζω*_r_*ω, to the closed-form voltage and power

(13) $$V\left(\omega \right)=\frac{\sum_{r}j\omega \;\theta_{r}\Gamma_{r}/D_{r}}{j\omega C_{p}+1/R+\sum_{r}j\omega \;\theta_{r}^{2}/D_{r}},\;\;P=\frac{{\left|V\right|}^{2}}{2R},$$

whose denominator is the electrical admittance plus the motional admittance of the structure. The term θ*_r_*^2^ is the electrical damping that shifts the loaded peak away from the natural frequency. Stress is recovered from the same solution. The station stress is with modal amplitudes η*_r_* = (Γ*_r_* + θ*_r_V*)/*D_r_*

(14) $$\sigma \left(x\right)=E_{s}\;c\left(x\right)\left|\sum_{r}\eta_{r}\;{\phi_{r}}^{\prime \prime }\left(x\right)\right|,$$

where *c*(*x*) is the critical-fiber distance of the station’s own section, the thinned face for the band, the net section for the through-cut and the rib underside for the pocket, maximized over the treated zone.

The results are given as four derived quantities. The mass-normalized first mode, the electromechanical figure of merit, the coupling regime parameter, the impedance-matched load and the mechanical floor of the half-power width are

(15) $$k^{2}=\frac{\theta_{1}^{2}}{C_{p}\;\omega_{1}^{2}},\;\;\frac{k^{2}}{4\zeta },\;\;R_{\mathrm{opt}}≃\frac{1}{\omega_{1}C_{p}},\;\;\Delta f_{h}\;\geq \;2\zeta f_{1}.$$

For the devices in this study *k*^2^/4ζ = 0.45–0.59, well into the weak-coupling regime where a single optimal load exists, *R_opt_* ≈ 86 kΩ; the half-power width exceeds its mechanical floor by the electrical damping the load injects. Finally, the protocol that names the paper is itself one equation: every treated design must satisfy

(16) $$f_{1}^{\mathrm{sub}}\left(f_{x},f_{z},\beta \right)=f_{1}^{⋆}=44.885\;\mathrm{Hz},$$

where *f*_1*,sub*_ is the fundamental of the substrate alone (patch removed, three-segment beam of Equation (9)) solved for the one free parameter of each topology (*β* for band and pocket, *f* for the through-cut) by bracketed root finding. Matching *f*_1*,sub*_ is matching root compliance rather than a mixture of stiffness and mass, for a reason specific to where the treatment sits. The fundamental obeys *f*_1_^2^∝k_1_/m_1_ with modal stiffness k1=∫EI φ1″2dx and modal mass $m_{1}=\int \mathrm{\varrho A}\varphi_{1}^{2}\mathrm{dx}$; the treated zone lies at the root, where φ_1_ is the smallest and φ_1_″ largest, so the removed material enters m_1_ at second order and k_1_ at first. Solving the substrate-only beam for the three matched designs makes this quantitative: the zone mass per unit length differs by 43% between the lightest (pocket) and the heaviest (band) root, yet the treated zone contributes only 0.6–0.8% of the modal mass. The zone bending stiffness each design must carry to reach 44.885 Hz agrees to 0.3% (0.461, 0460 and 0.460 of the solid section for band, through-cut and pocket), as does the static tip compliance under a unit end load (3.456, 3.462, and 3.464 mm N^−1^ against 2.00 for solid beam). Equal substrate-only fundamental is, therefore, equal root compliance to better than 0.3%. The residual device-mass differences reported in Section 5.4 are consequences of the treatment, not defects of the control, and Section 5.7 examines them through mass-normalized power and matched-device-resonance frame, both of which preserve the ordering.

## 3. Finite Element Model

The high-fidelity reference was constructed as a 3D coupled-field model in ANSYS Mechanical APDL (Table 1). The substrate uses quadratic structural tetrahedra and the piezoceramic quadratic coupled-field tetrahedra that carry all the anisotropic elastic, piezoelectric and permittivity data; a circuit resistor links the equipotential electrode sets. The bond between the patch and the substrate is conformal by construction: The patch is meshed independently and bound to the substrate surface by interface constraint equations, so that over a through-hole where there is no substrate, the patch nodes are simply left free, naturally representing the local loss of composite action. The tie was cross-checked to a shared-node (glued) formulation on the band device: the two independent bonding formulations agree in frequency, voltage, power and stress to 0.01%. Every run is self-checking: the script aborts with a diagnostic on any failure, checking the constructed volume against the exact expected value (<0.2%), the centroid location, the constraint-equation count, and the patch-engagement frequency shift. Each analysis contains a modal solve, a full harmonic sweep about the fundamental storing only degrees-of-freedom histories, and a second single-frequency harmonic at the identified peak storing the full field for stress recovery.

**Figure 3:**
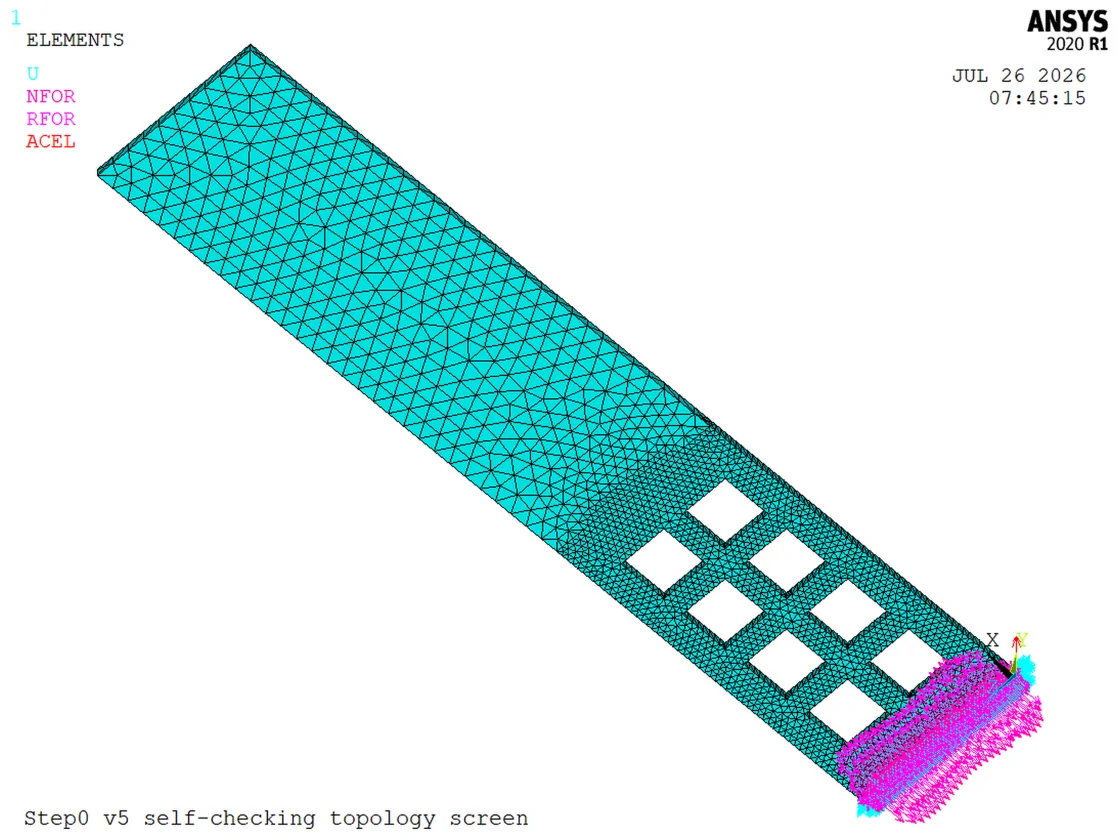
Finite element mesh of the through-cut screening model generated by the self-checking deck (ANSYS 2020 R1). The symbols of the clamped-face constraint and the base acceleration are displayed.

**Figure 4:**
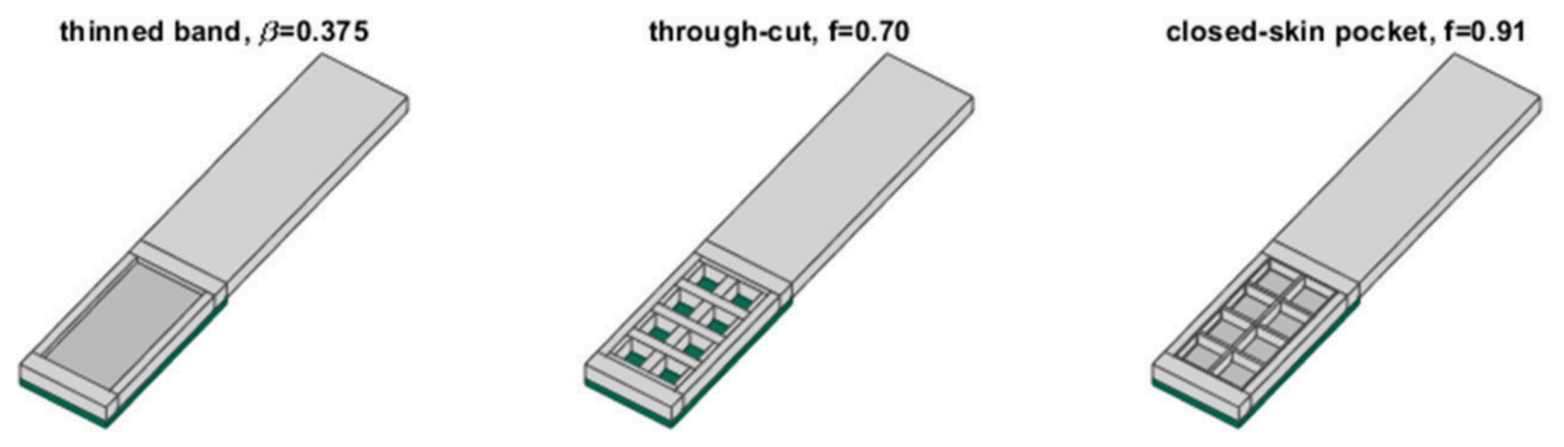
The three coupled analysis geometries as built, rendered from the parameterization of Figure 1 with the treated face toward reader and piezoceramic patch (teal) beneath the root the thinned band (β = 0.375), the through-cut (f = 0.7), whose openings expose the ceramic at their floors, and closed-skin pocket (f = 0.91), whose skin closes them. Thickness exaggerated for visibility.

Figure 3 shows one of the models as ANSYS builds it, showing the swept mesh and symbols for clamped face constraint and base-acceleration load. It is typical of all runs in the campaign: no geometry continues to solution without passing the volume, centroid and constraint-count self-checks in Table 1. The three analysis geometries as built are shown in Figure 4; their first two mode shapes are given in Figure 2. The mode-shape plot is the whole study mechanism in one line: in every device, the fundamental concentrates its curvature inside the treated zone beneath the electrode, the shaded band, which is exactly why softening that zone amplifies the harvested strain, and why the three devices, identical outside it, order themselves by how efficiently each topology converts that shared curvature. The through-cut has the highest frequency, as the full-thickness ligaments, stiffened further by the bonded patch, provide the stiffest composite root. The pocket has the lowest because the thin skin provides the least stiffening. The second harmonic of the sweep grazes mode two, which has its node near mid-span and is a factor of ∼5.8 above the fundamental for every design, safely outside the harvesting band. The finite element stress fields tell a consistent spatial story without contour plots: the maximum over the whole treated zone coincides with the maximum over the first feature row alone, to every printed digit in the band and through-cut devices. The critical material is situated in the first row of ligaments (through-cut) or in the first step (band), exactly where the net-section and plate-formula arguments of Section 2.2 place it. The pocket’s peak sits on the rib undersides, at the shifted neutral-axis arm.

## 4. Model Validation Against Three-Dimensional Finite Elements

The analytical chain was validated link by link against independent three-dimensional finite element computations, working upwards through the same hierarchy in which it was developed: first section mechanics, then the matched-compliance frequencies, then the complete coupled devices.

At the level of section mechanics, the band Formula (1) reproduces the finite element peak stress under the screen loading to 0.9% (4.57 vs. 4.61 MPa). The net-section bound (5) correctly locates the critical ligament of the through-cut and underestimates its magnitude, the excess quantifying the corner and width-wise shear-lag concentration of square features; the sharpest test is, therefore, the pocket/through stress ratio, in which those common effects cancel, and, there, the composite-section prediction of 1.22 stands against the finite element 1.19. 

Moving up to the matched-compliance frequencies, solving the segmented beam for the design parameter returns the reference substrate fundamental (44.885 Hz), reproducing the finite element matching points to −2.2% (band, *β*: 0.367 vs. 0.375), +1.1% (pocket, f: 0.919 vs. 0.909) and +8.4% (through, where the series idealization binds). The inverse finite element band datum implies a stiffness ratio of 0.461, versus 0.456 for the railed composite and 0.244 for the plate bound. The solid side margins are fully effective for global stiffness, while the local surface stress is still plate-like. These are two regimes, each pinned by its own finite element benchmark. 

With the patch present, the model predicts the three fundamentals within 4.5% for the complete devices (67.9/74.7/65.7 vs. 70.9/75.7/67.5 Hz; the untreated device 83.5 vs. 86.9). The absolute powers for the fully bonded topologies lie below the three-dimensional values. The familiar distributed-parameter offset, and the offset being common, cancel in ratios: the pocket-to-band power ratio is predicted to 2.6% (0.903 vs. 0.927). For the through-perforated device, the two bonding idealizations in Section 2.3 give 8.6 µW (rigid-α) and 16.9 µW (force-continuity) in the model frame. These bounds are compared with the finite element only after the common offset is removed, and that offset is calibrated on devices in which bonding is unambiguous: the finite element to model power ratio κ is 1.31 (solid), 1.32 (pocket), and 1.32 (finite element powers from Table 2). Carried into the finite frame, the through-cut bracket becomes 11.4–22.4 µW, and the finite element results of 20.7 µW lie inside it, 84% of the way toward the force-continuity bound; the smallest of the three calibrations (1.31) still places the upper bound at 22.0 µW, above the finite element value. The ceramic spanning each hole, therefore, behaves as largely active rather than inert, an effective coverage of roughly 0.9 against the geometric 0.61 of the rigid models. The tie provides independent corroboration for the coverage fraction: the conformal bond produced 16,863 constraint equations for the through-cut as opposed to 23,220 for the band at equal mesh density, a bonded fraction of 0.73 versus the whole-electrode 0.686, and the untreated device gives the same 23,220, confirming the full-face baseline. The full model was also implemented independently twice (Python 3.14 and MATLAB R2021a) with agreement to all printed digits.

The correction is single scaler per device, κ = P_FE_/P_model_, evaluated at the matched design and 1 MΩ. It is not a fitted parameter: it is measured on devices whose bonding is unambiguous and applied to the one device whose bonding is bracketed. Table 3 collects it, with frequency and stress errors, for every topology and for three further designs from inside the optimization range that were run in the coupled finite element for this purpose (Section 5.5). Across these six anchors, which span solid, thinned, deeply thinned, profiled, perforated and pocket roots, κ ranges from 1.28 to 1.36, within ±3% of its mean and rising gently with the depth of the cut. The offset is also load-dependent: the matched-load powers in Section 5.5 are within 7–11% of the finite element, and the larger 1 MΩ offset is a property of the open-circuit branch, where the response is coupling-dominated. The offset enters no optimization in this study: every landscape, sweep and profile search compares design within the model frame, where a common factor cancels, so its only role is to place the through-cut bracket and finite element anchors on one axis.

**Figure 5:**
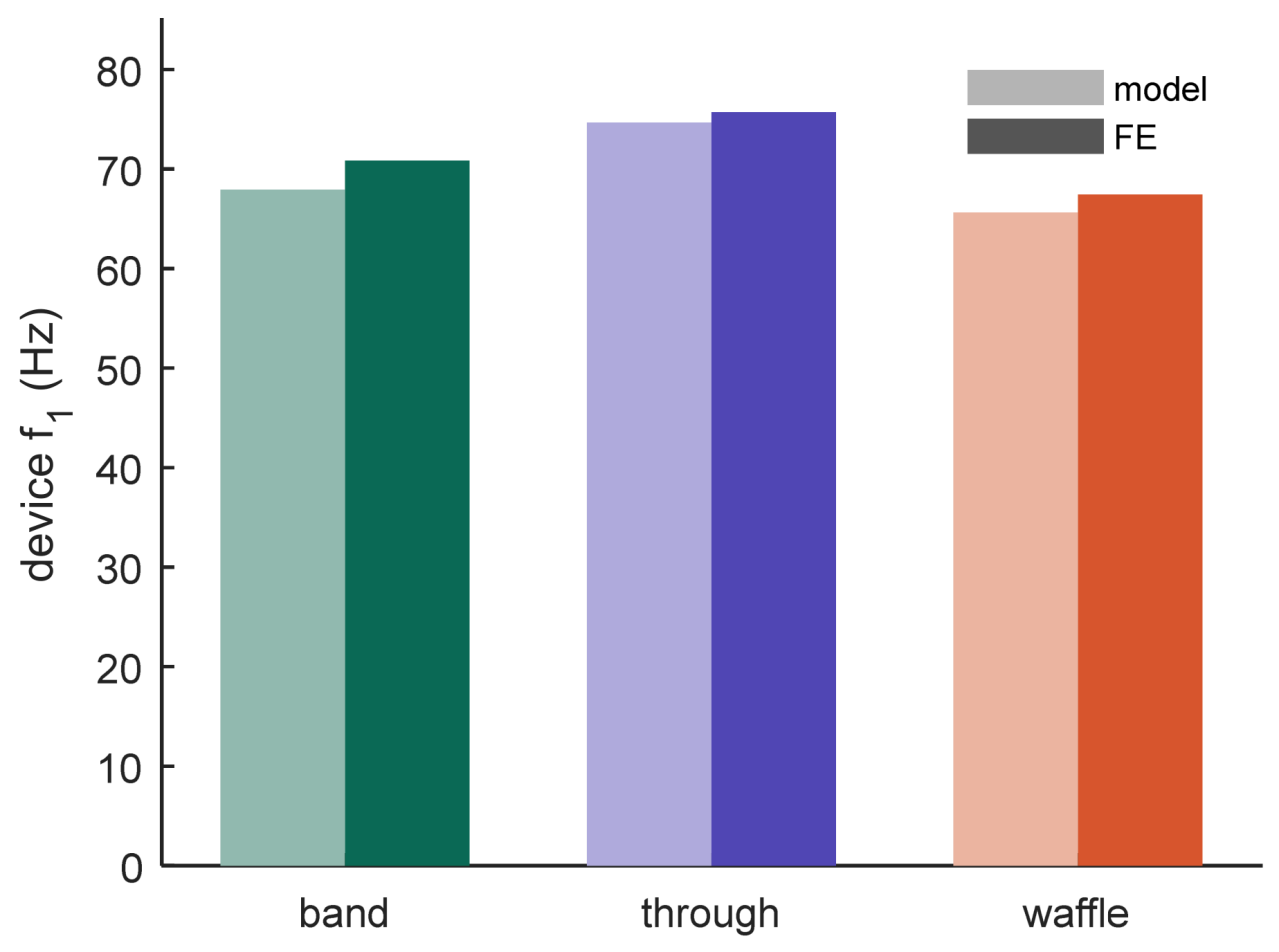
Device fundamentals *f*_1_. Model vs. finite element at the three design points.

Figure 5 summarizes the validation in one glance: model and finite element to a few percent for all three devices, and the agreement is a property of the quantity being compared. The fundamental is the square root of a Rayleigh quotient, global bending stiffness weighed against global inertia with the mode shape as the weighting function, so every local detail the beam model simplifies away, hole corners, three-dimensional strain fields, the load path around a pocket wall, enters the frequency only through its integrated contribution to *EI*(*x*) and *ρA*(*x*) along the span. Two facts then compress the error: the square root halves it, the Rayleigh quotient is stationary at the true mode, so the first-order error in the mode shape produces only a second-order error in the frequency. The residual bias, model a few percent below finite element for every device, is the signature of the treated-zone homogenization of Section 2.1, a section-level simplification, not a failure of the electromechanical coupling.

## 5. Results and Discussion

The excitation is 1 m s^−2^ unless otherwise noted, R = 1 MΩ, and ζ = 0.02; mode-shape figure and the validation checks use the undamped natural frequencies, the power results the frequency of the damped peak. Throughout, values labelled (FE) are three-dimensional finite element results and values labelled (model) are from the segmented model; comparisons within a frame are exact, and comparisons across frames carry the offset of κ in Section 4.

### 5.1. The Conventional Comparison: Frequency Free

**Figure 6:**
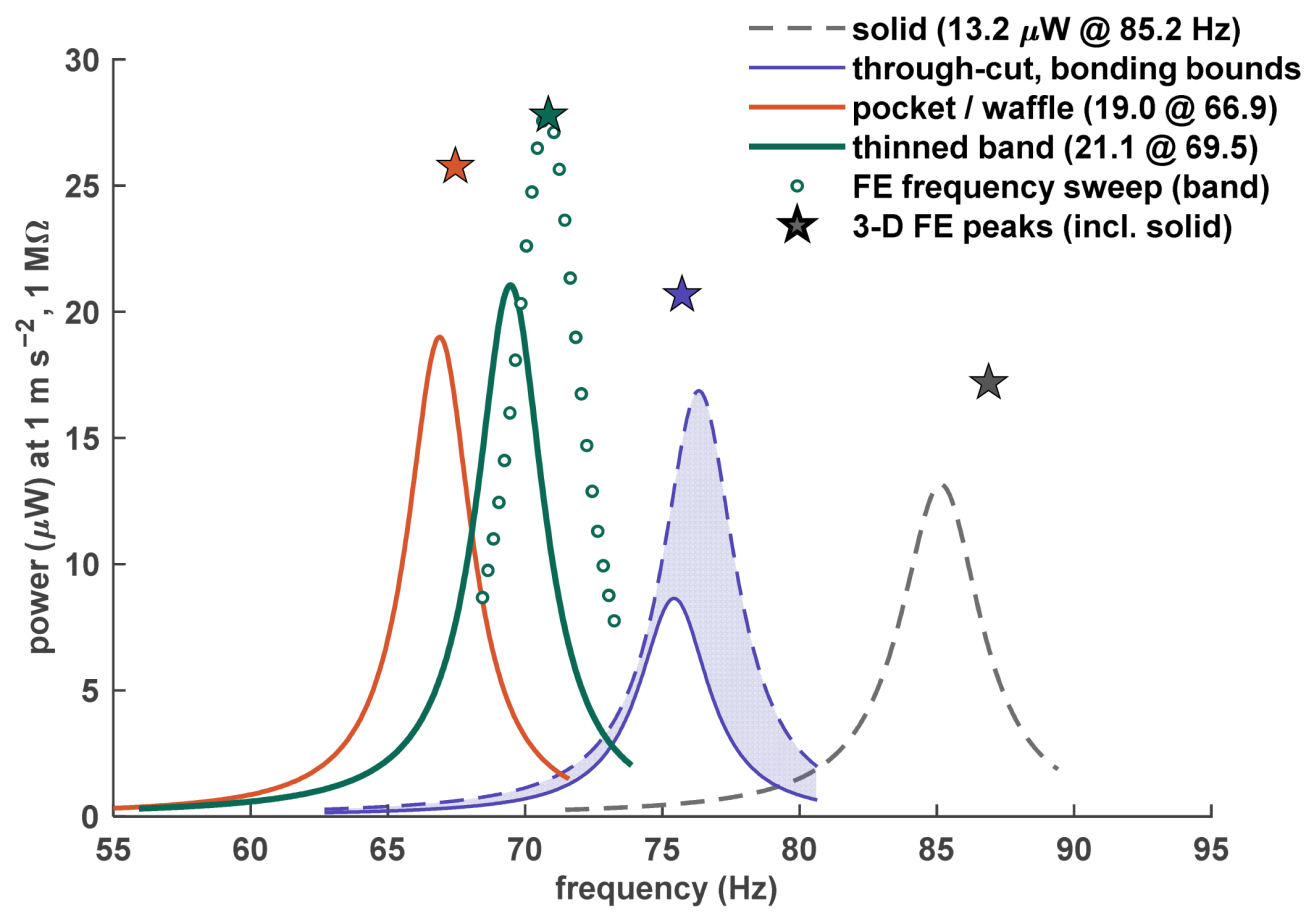
Unconstrained power frequency responses of the four devices at 1 MΩ.

At 1 MΩ, every root treatment (model) outperforms the solid beam and usefully moves the resonance downward. The familiar cellular-substrate picture is reproduced. Against the solid device, the finite element frame gives the following: band +62%, pocket +50% and through-cut +20% (20.67 against 17.19 µW), squarely inside the 10–25% band the cellular literature reports for perforated roots; the model frame gives +60% and +44% for the fully bonded pair with the through-cut between its bonding bounds. In this conventional frame, the twist is already visible: the no-hole band is on top of the field. Figure 6 has two aspects that need a physical interpretation. The finite element peaks are uniformly offset above the model curves because a three-dimensional solid is more compliant than a Euler–Bernoulli beam of the same section. Transverse shear and local deformation around the treated zone open deformation paths that plane-section kinematics cannot represent, and that extra compliance lets the same base excitation drive more strain into the patch at resonance. The offset is common to all designs and cancels wherever we compare them to one another. And the pocketed root peaks below the band, although it resonates lower, which at first seems backward: softer should be stronger. The reason is the coupling arm. The stiff skin of the pocket pulls the neutral axis toward the ceramic, so each unit of curvature puts less strain on the transducer. Its extra compliance buys curvature and then spends part of the purchase on a shorter lever. For the band, we went one step further and exported the full harmonic frequency-response sweep of the finite element, plotted as the open dots in Figure 6. It reproduces the harvested peak to the final digit, 7.455 V, 27.79 µW at 70.85 Hz, and traces a half-power width of 3.1 Hz against the purely mechanical 2*ζ f*_1_ = 2.8 Hz expected at this near-open-circuit load, the small excess being the residual electrical damping. The three-dimensional solution, therefore, confirms the shape of the response, not merely its peak. Between the model curve and finite element dots in Figure 6 is only the uniform vertical offset already accounted for.

**Figure 7:**
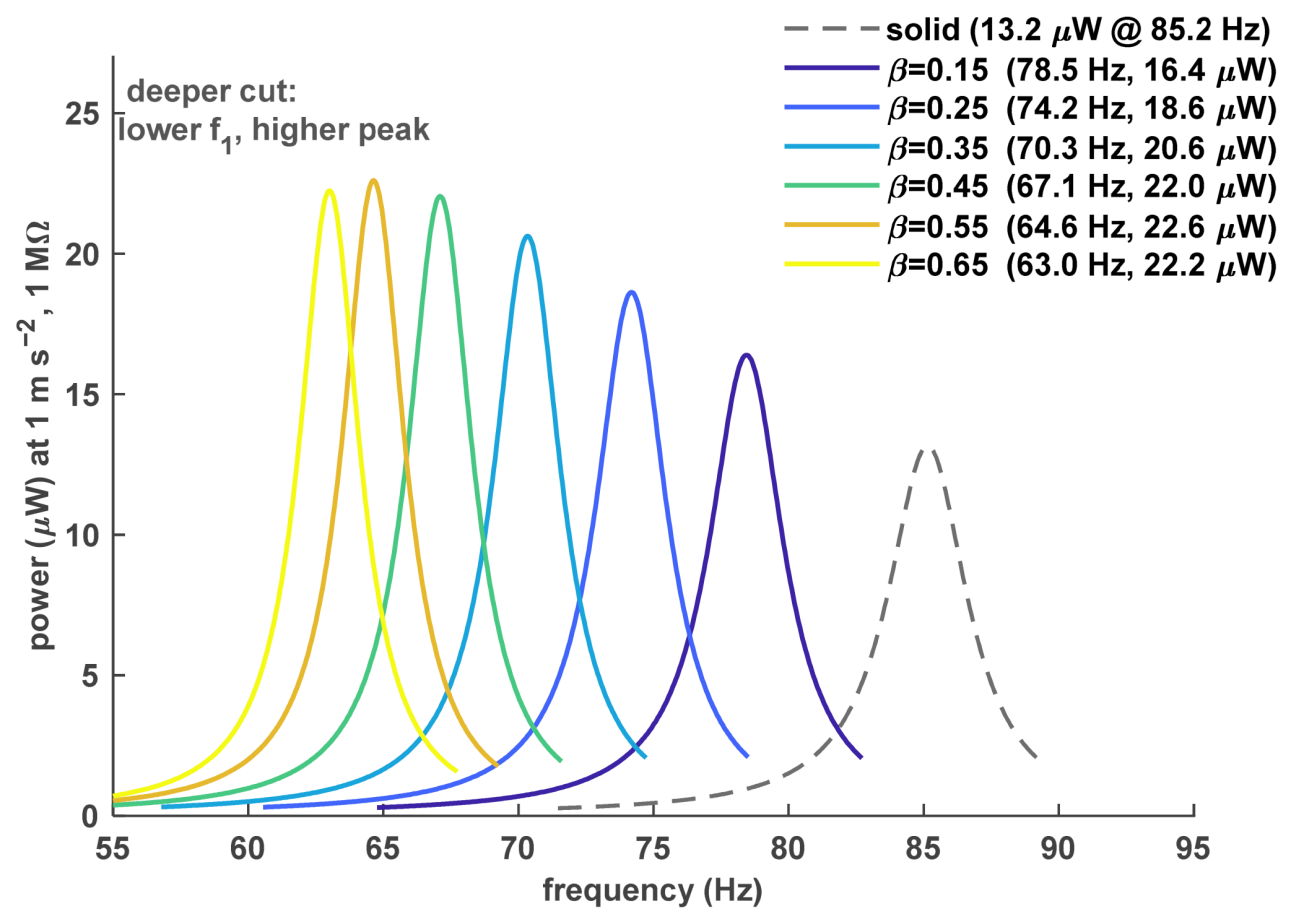
Depth sweep of the uniformly thinned band vs. solid β ≈ 0.55.

Figure 7 shows the band’s single design freedom, depth, which rises to a shallow optimum at β ≈ 0.55 (+72% over the solid beam), while the resonance falls some 20 Hz with no added mass, before the curve turns over as coupling dilutes. One milling depth, thus, covers the same power-frequency design plane that cellular papers cover with cell size. The turnover in Figure 7 is worth pausing on, because it is the depth axis in miniature: as the cut deepens, compliance and, therefore, curvature keep increasing, but the neutral axis of the thinned section also lifts toward the patch, shrinking the coupling arm *h_pc_*, and modal mass leaves the root. The first effect raises the response; the other two penalize the conversion. Their product peaks close to *β* ≈ 0.55 and drops gently beyond it, so the last twenty percent of achievable softening costs nothing electrically and quite a lot of stress.

### 5.2. Multivariable Optimization of the Hole Dimensions, Frequency Free

**Figure 8:**
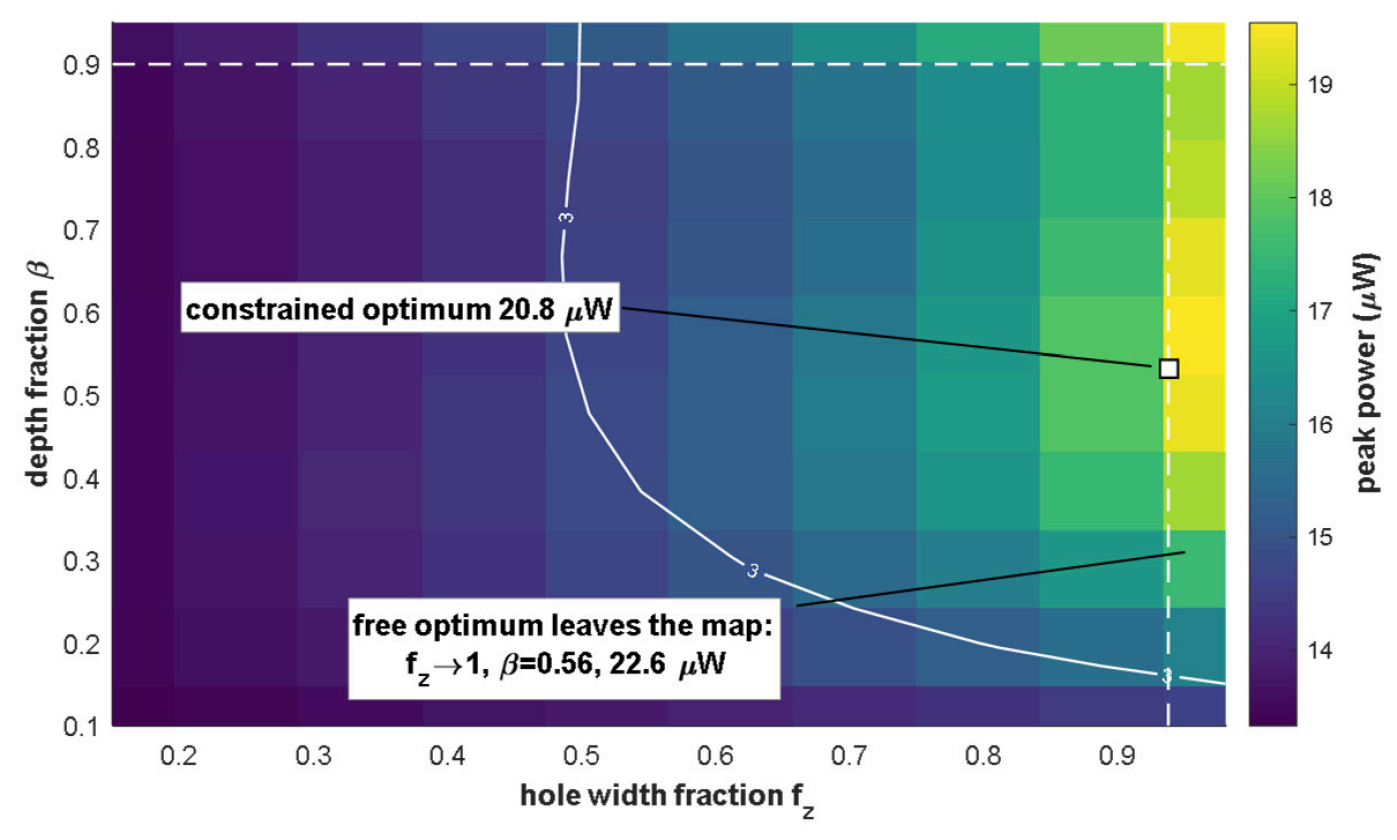
Frequency-free peak power over hole width and depth at *f_x_* = 0.70, with (white) and the manufacturing limits (dashed). The constrained optimum is on the ligament limit, the free optimum leaves the hole family altogether.

**Figure 9:**
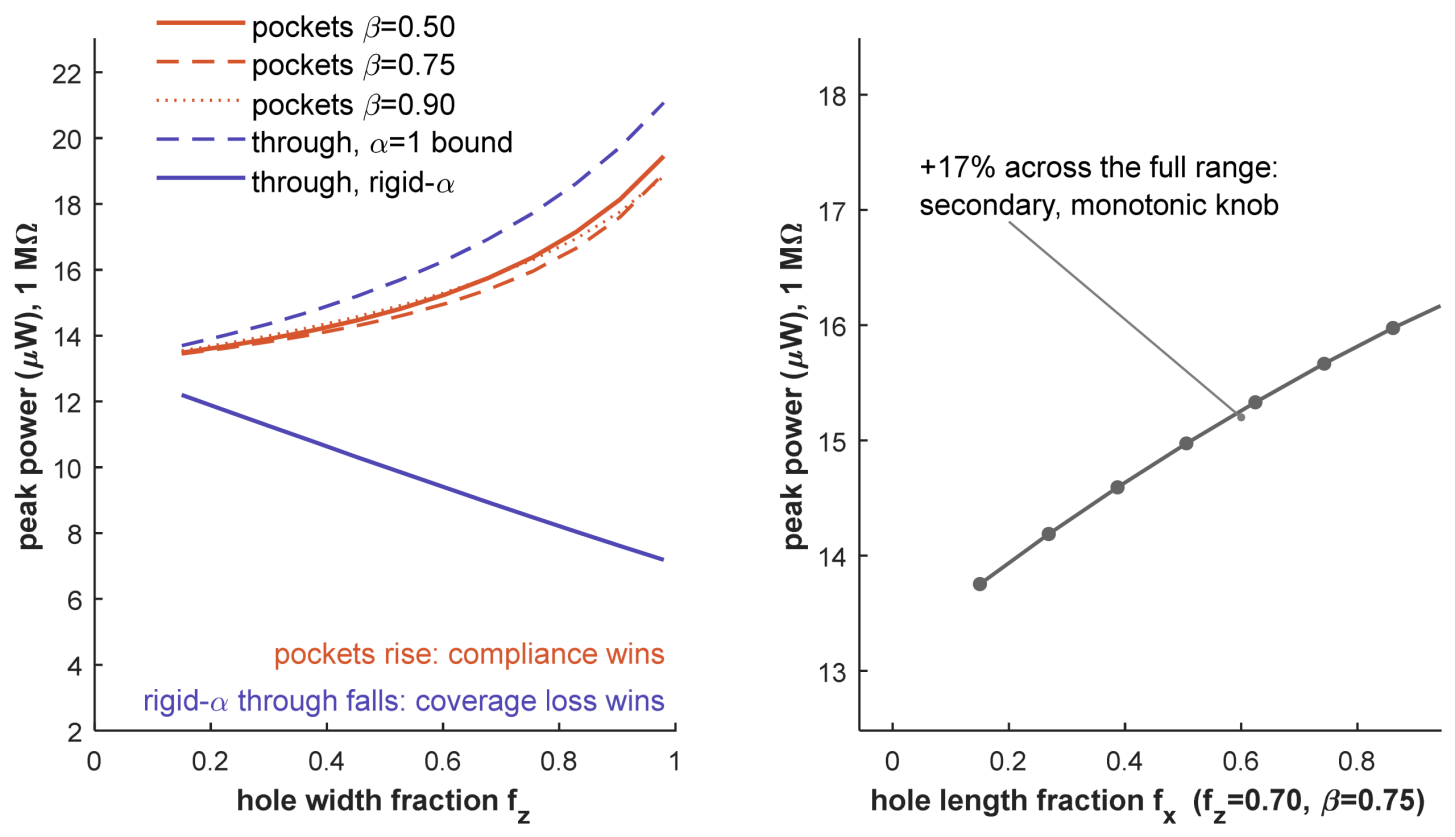
Frequency-free hole dimension sweeps at 1 MΩ left. vs. hole width fraction *f_z_*, compliance drives pockets up, coverage loss drives rigid-α through-cuts down. Right: the along-beam fraction *f_x_* is a secondary monotonic knob.

The hole dimensions were optimized simultaneously over all three variables (*f_x_*, *f_z_*, *β*) in the frequency-free frame, with the load re-optimized inside every candidate; the one-variable sweeps in Figure 7, the two-variable map in Figure 8 and the sweep in Figure 9 are diagnostic sections through this landscape, shown to expose the mechanisms, not the search itself. The search is a box-clipped Nelder–Mead ascent from (0.6, 0.6, 0.5) with *x_tol_* = *f_tol_* = 5 × 10^−3^ over 0.15 ≤ *f_x_, f_z_* ≤ 0.999 and 0.05 ≤ *β* ≤ 0.999. A_n_ independent differential-evaluation search over the same box converges to the same wall optimum (*f_x_ = f_z_* = 0.999, β = 0.557, 22.57 µW, model), so the boundary result is not an artifact of the local method. The depth gain is also confirmed by the coupled finite element: the deep band β = 0.557 delivers 30.7 µW at 1 MΩ against the matched band’s 27.8 (FE, +10% model +7%; Table 3). Each candidate (*f_x_*, *f_z_*, *β*) is evaluated by the validated segmented model with a modal solve, coupling at the appropriate active coverage and bounded scalar search for the peak |V|^2^/2R; the ascent converged in about 100 evaluations. Two constraint sets are considered: bounds only (*fx*,*fz* ≥ 0.15, *β* ≥ 0.05) and a manufacturing set adding a minimum closing skin (1 − *β*)*hs* ≥ 0.1 mm and a minimum ligament (1 − *fz*)*pz* ≥ 0.5 mm.

The underlying sweeps are shown in Figure 9. For closed-skin pockets, the compliance effect dominates: power increases monotonically with both hole dimensions (+41% over the width fraction, +17% along the beam), so every pocket is driven to the merge limit. For through-cuts, the two mechanisms compete: under the classical rigid-α assumption, coverage loss wins and power falls as the holes grow, whereas under the force-continuity bound, the compliance gain dominates mildly. As the finite element sits close to the latter, real through-holes gain weakly at best. Even the optimistic bound is below the band limit. The opposing slopes in Figure 9 have a neat origin. The skin keeps all the electrode working for pockets, so the only active mechanism is compliance, and the curves can only rise. For through-cuts, each increment in hole width does two things simultaneously: it softens the root a little, and it converts the bonded electrode directly into a bridge, whose contribution under the rigid assumption is zero. The coverage term decreases linearly in the footprint, while the compliance term saturates, so the classical model correctly predicts, within its own assumptions, that bigger through-holes are worse. The along-beam fraction (right panel) moves only though the series average of the section compliance and so is a gentle, monotonic, secondary knob. The free optimum terminates, once again, hole-free: *fx,fz* → 1 with *β* = 0.558, numerically identical to the plain band’s own depth optimum in Figure 7 (22.6 µW, +72% over solid). The best design with holes under the manufacturing caps (*f_z_* = 0.938, *β* = 0.53) is 8% behind the plain band (Figure 8). Color climbs smoothly up the width axis and only gently along depth, the brightest cells hugging the right edge: power wants the widest pocket available at a middling depth, for the same compliance versus lever arm reasons as the depth sweep. The white stress contours cut diagonally across that trend (wide and deep is where the ribs thin and the fiber arm grows) so the two surfaces disagree about which corner to prefer. The most telling thing is where the constrained optimum falls: at the ligament limit, not at some interior sweet spot. A_n_ interior optimum would have been the hole’s one argument for existing; a wall optimum is the map’s way of saying the search wanted to continue until the holes were gone. The no-hole conclusion is, therefore, not an artifact of the matched-compliance protocol. With the frequency completely free, the optimum of the family still has no holes. One nuance carries over to the profile study: the unconstrained power optimum sits deeper (*β* ≈ 0.55), at about 2.5-times the stress of the matched design (*β* = 0.375); power and durability trade along the depth axis, and the choice between them is an application decision, not a topological one.

### 5.3. The Matched-Compliance Screen

**Figure 10:**
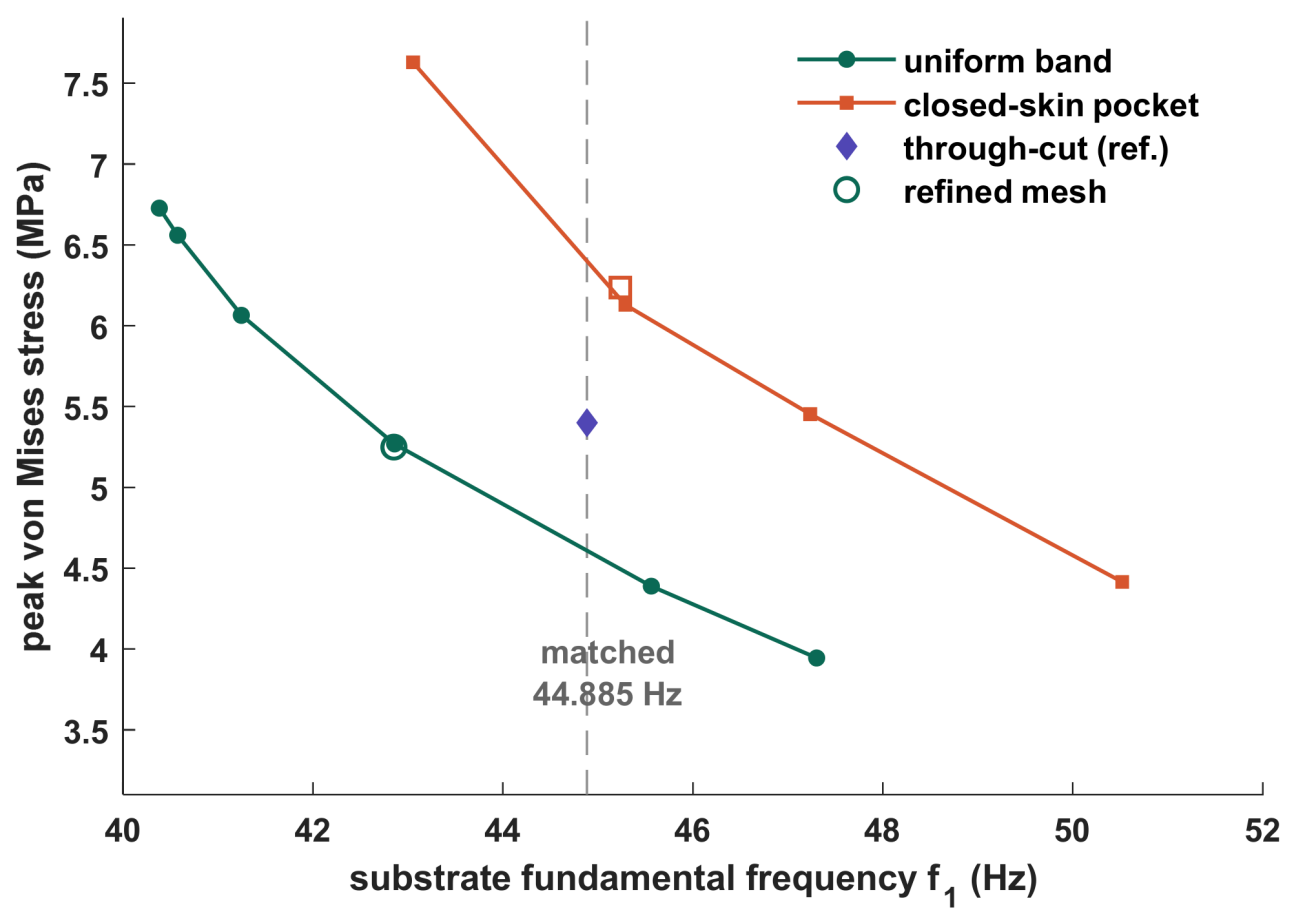
Substrate-only screen: peak stress vs. fundamental frequency for the two continuous families and the through-cut reference. Open symbols are refined-mesh checks. Vertical line: the matched compliance where all devices are compared.

Figure 10 shows the substrate-only screen that defines the protocol: peak von Mises stress vs. fundamental frequency for the thinned-band and blind-pocket families, with the through-cut reference. Reading down, the matched frequency gives the main mechanical result: at equal compliance, the band carries 4.61 MPa, the through-cut 5.40 MPa (+17%) and the pocket ≈ 6.4 MPa (+39%). The ordering is stable with mesh refinement (<2% shifts) and for two independent element formulations (hexahedral and tetrahedral models agree to 1.5%). Each margin is explained by the closed forms in Section 2.2: the net-section factor for the through-cut, the adverse neutral-axis shift for the pocket. All three families are on declining curves because softening always trades stress for frequency, but they decline at different rates. The band pays the gentlest price because a plain thin section retains its full width and a symmetrical fiber distance. The through-cut channels the same bending moment through ligaments, which carry it on a fraction of the width; and the pocket, stiff skin on one face and ribs on the other, shifts its neutral axis toward the skin so that the rib bottom is working at the longest lever arm in the field. The refined-mesh points confirm this is mechanics, not discretization. The peaks reported are on first-row ligament faces next to corners (through-cut, band step) and on rib undersides (pocket) because the features are square-cornered. The corresponding singularity-free nominal values, from the net-section and composite-section formulas, are 4.3 MPa (through, corner factor ≈ 1.25 at this mesh) and ≈5.3 MPa (pocket, ≈1.2) compared to the band’s 4.6. On the nominals, the verdict on the nominals is split: the penalty on the pocket survives (5.3 to 4.6), but the band–through gap does not. The gentler root is the through-cut on section nominals, 4.3 vs. 4.6 in the screen and near parity in the coupled frame (5.70/1.25 ≈ 4.6), because thinning magnifies section stress by 1/(1 − *β*)2 = 2.56, while the through-cut’s net-section factor is only *b*/*bn* = 2.27. The band’s stress advantage over the through-cut is at the square-cornered peaks, exactly the places a filleted device would soften; the pocket-worst conclusion and all power results are unaffected.

### 5.4. Three Harvesters at Matched Compliance

**Figure 11:**
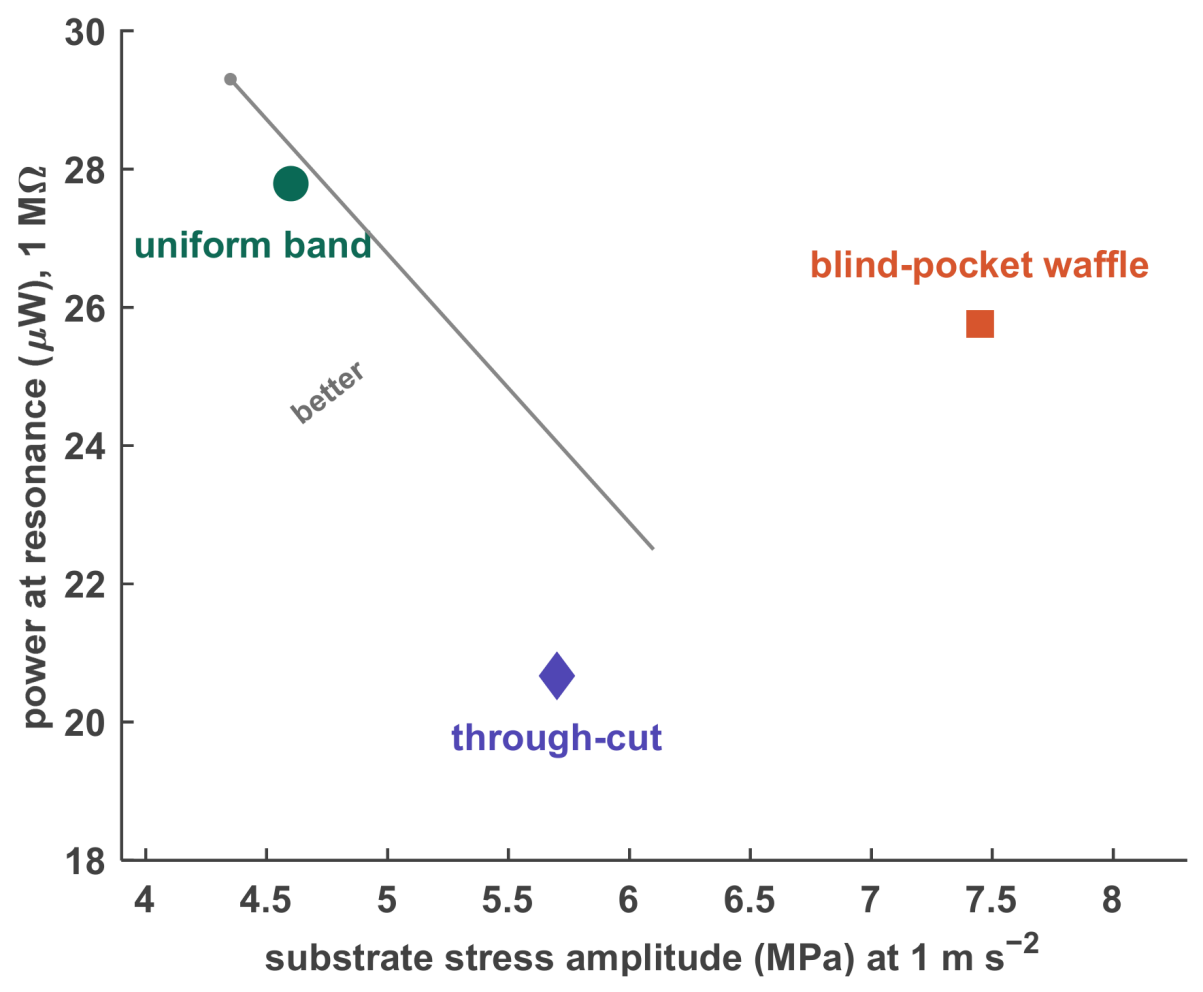
Power vs. substrate stress for the three matched devices: the thinned band alone is at the favorable corner.

Table 2 and Figure 11 show the coupled results. The uniformly thinned band dominates both axes simultaneously: 34% more power than the through-perforated root at 19% lower peak substrate stress, and 8% more power than the closed-skin pocket at 38% lower stress. The band harvests 1.7× any cellular design per unit of peak stress amplitude. The pocket out-powers the through-cut (its unbroken skin keeps the patch fully coupled) but suffers the worst stress in the field, exactly as the neutral-axis analysis requires; the through-cut’s power deficit is the direct signature of its unbonded electrode fraction (Section 4).

For comparison with the standard baseline, the same model gives *f*_1_ = 83.5 Hz for the untreated solid device, and the coupled finite element gives 17.19 µW at 86.9 Hz. The segmented model’s 13.2 µW is 23% below it, the same offset family as the treated devices, so the model-to-finite-element transfer assumed earlier is now validated rather than assumed: the through-perforated root gains +20% over the solid beam, squarely in the 10–25% band the cellular literature reports.

Figure 11 collapses the table to the plane a designer would optimize. The most telling feature of the figure is what is missing: a trade-off line. In an unconstrained comparison, the three devices would string out along the usual softness-for-stress frontier, but the matching has already spent that trade equally for everyone, so the scatter now shows only how each topology converts the shared compliance budget. The band, with its full-width section and unbroken electrode, sits alone in the favorable corner because it wastes none of it. The through-cut is low: it burns part of the budget on ceramic bonded to nothing and pays net-section stress for the rest. The pocket is an efficient converter, but it works at the longest lever arm in the field, which is why it climbs in power and slides right in stress at the same time. The plot shows dominance, not a trade-off. The same ordering holds at matched device frequency, at optimal load, per gram and as normalized power density (Table 4, Section 5.7).

### 5.5. Exhaustive Search of the Design Space

Under the matched-compliance constraint, three coordinated searches generalize the three-design comparison to the whole family: a global search over the hole footprint with depth solved per point for matched compliance and load optimized, a constrained search over thickness profile, and the load sweep. Each was run as an exhaustive map plus multi-start local refinement, cross-checked with an independent differential-evaluation search that converged to the same boundary optimum (*f_x_* = 0.999, *f_z_* = 0.981, 59.97 µW, model), and Table 5 specifies every search’s variable, bounds, constraints, algorithm and stopping criteria.

**Figure 12:**
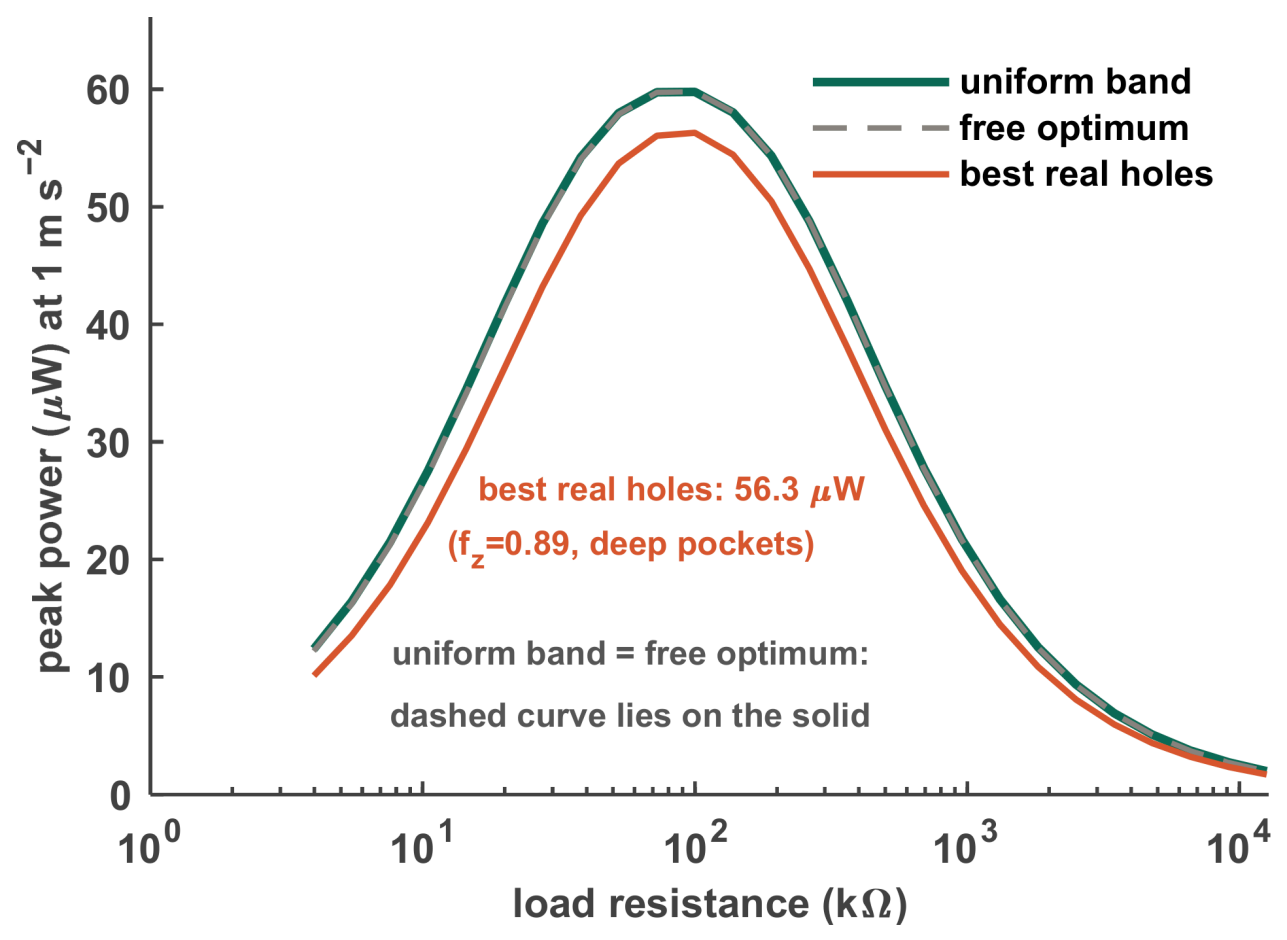
Power versus load for the uniform band, the free optimum (indistinguishable) and the best design with real holes.

**Figure 13:**
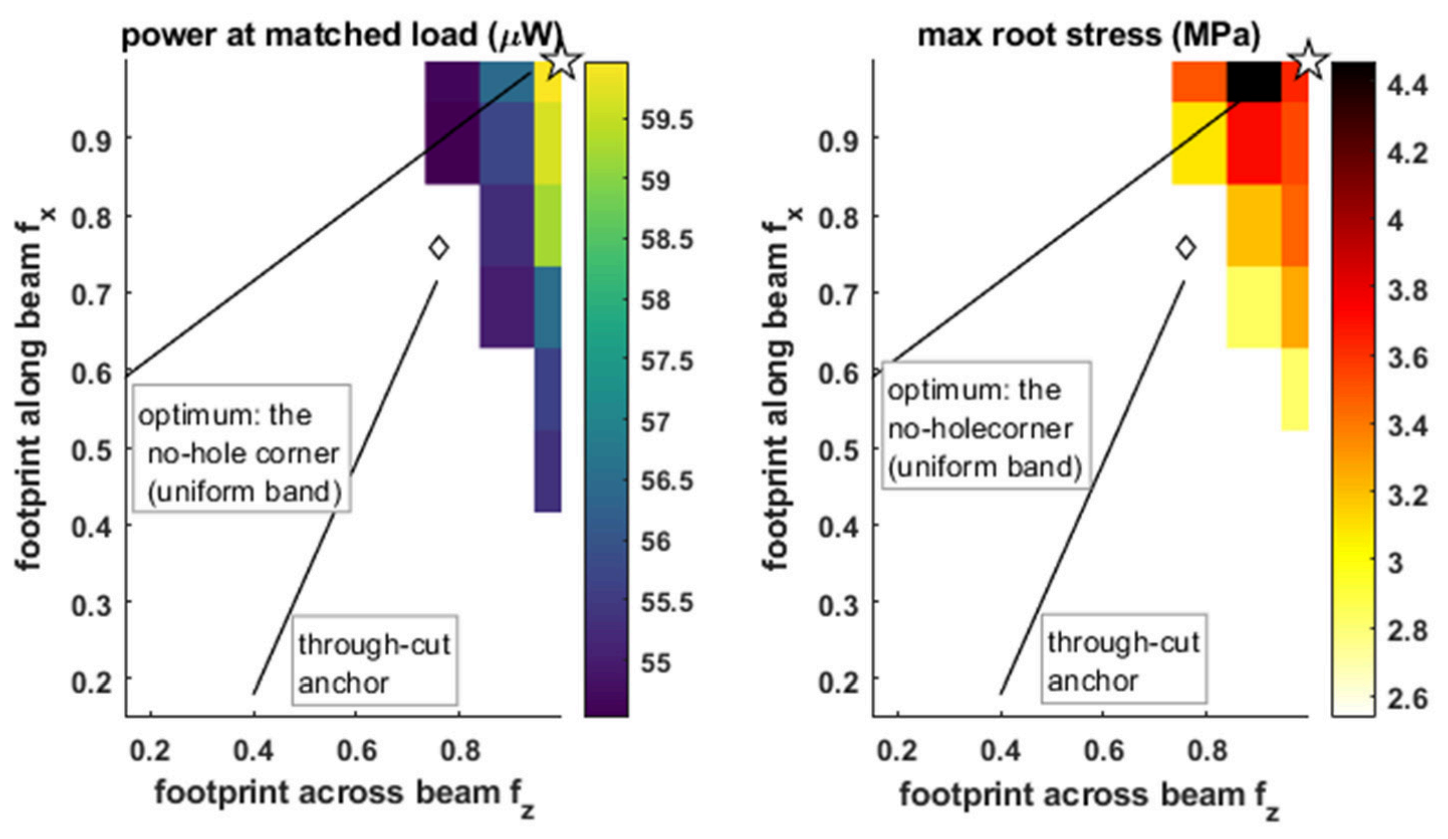
Matched-compliance landscape over the hole footprint: power at matched load (**left**) and maximum root stress (**right**); the depth of each cell is solved for the reference compliance. The star marks the optimum at the no-hole corner, f_x_, f_z_ → 1 (the uniform band); the diamond marks the through-cut anchor, f_x_ = f_z_ = 0.76. Blank cells are footprints that cannot reach the reference compliance within the substrate thickness (β ≤ 1). The unpunched corner is the best.

Hole geometry: With free footprint fractions (*f_x_*,*f_z_*), depth per design for matched compliance and load optimized, an 81-point landscape (grid spacing ≈ 0.106 per footprint fraction) plus local refinement (Nelder–Mead, tolerance 5 × 10^−3^) converges to the corner. Five random-restart ascents in the frequency-free frame terminate at the same point, *β* = 0.556 ± 0.006, so the boundary optimum is not a start-point artifact. The search converges to the corner *f_x_*,*f_z_* → 1, the configuration in which the holes merge and disappear into the uniform band. The best design with real holes (*f_z_* = 0.89, deep pockets) gives 6% less power at 23% higher stress than the band for all loads from 4 kΩ to 10 MΩ (Figure 12). Its no-hole boundary (Figure 13) is the optimum of the perforation family.

The two panels in Figure 13 show why the search had nowhere else to go. The power surface is extremely flat, a few percent over all feasible, because the holes keep the compliance budget fixed, and at fixed compliance, the harvested power hardly distinguishes between the arrangements of the holes, and the very small gradient points steadily to wider, shallower features, which is the direction in which the holes go away. The stress surface contains all the structures missing from the power surface. It degrades as the footprint localizes, because a smaller hole at matched compliance must cut deeper, and deep narrow features concentrate curvature in slender ligaments. The objective is flat and the penalty is not, and so the landscape can only push the design to its boundary, and the boundary here is the no-hole corner. The same point is made along the electrical axis in Figure 12: band and “optimum” are one curve to within line thickness over four orders of magnitude of load resistance, and the best genuine-hole design runs parallel below them everywhere. The gap never closes because the load can only re-divide the same RC partition, identically for every design; a deficit born in the coupling survives any resistor. The landscape’s best design with real holes (*f_x_ = f_z_* = 0.893, depth solved to *β* = 0.892) was also run in the coupled finite element: 23.2 µW at 1 MΩ against the band’s 27.8 (FE, −16%), a wider deficit than the model’s −14%, so the no-hole conclusion holds the finite element with margin (Table 3).

**Figure 14:**
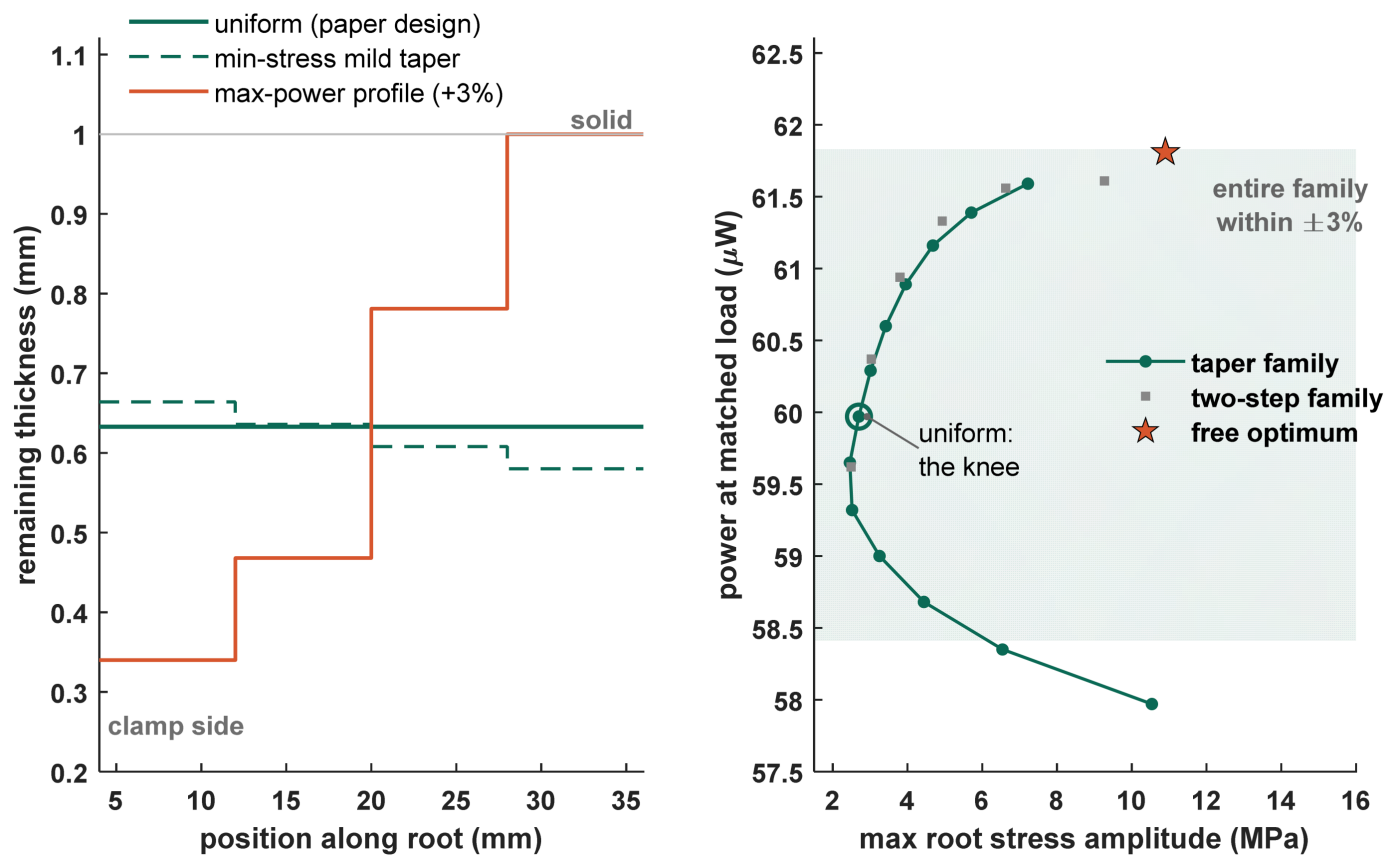
Thickness profile optimization. Candidate profiles (**left**) and power-stress plane (**right**). The whole family is ±3% in power. The uniform band is at the Pareto knee.

Thickness profile (Figure 14): Replacing the uniform band with the free four-segment profile (tapers, steps and an equality-constrained gradient search, Table 5) yields at most +5% in the model at matched load, with power-maximizing profile front-loading the depth (0.66, 0.66, 0.53, 0.22 of h_s_ removed, clamp side first). Because a gain of this size lies near the model’s ratio resolution, the profile was verified directly in the coupled finite element (Section 4, Table 3). The finite element confirms the gain and enlarges it: at matched load, the profile delivers 70.3 µW against the uniform band’s 64.4 (FE, +9.2%) and 30.1 against 27.8 µW at 1 MΩ (FE, +8.2%), so the gain survives at 1 MΩ. Its price is peak substrate stress 1.5–1.6-times the band’s (FE: 6.98 vs. 4.61 MPa at 1 MΩ, 4.84 vs. 2.99 MPa at matched load), a smaller penalty than the model’s fourfold result. The two candidates, therefore, mark the ends of one trade: the uniform band is the lowest-stress member of the family, the front-loaded profile the highest-power one, and both are thinned roots. Against the family’s best design with real holes, verified in the same finite element at 23.2 µW (Table 3), the uniform band leads by 20% and the profile by 29%.

**Figure 15:**
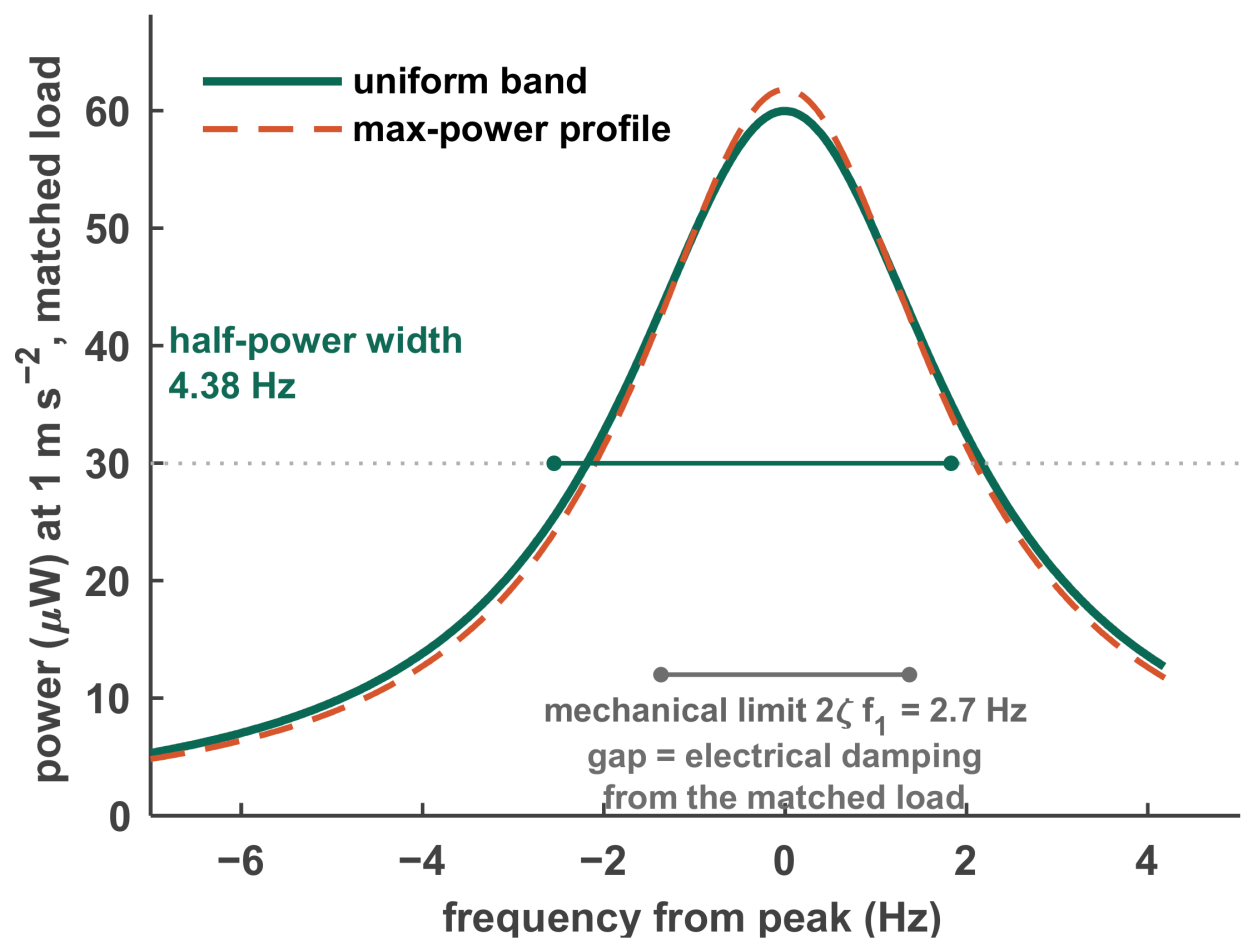
Resonance curves of the uniform band and the max-power profile at matched load: same half-power widths, set by mechanical + electrical damping.

Bandwidth*:* The maximum of the half-power width preserves the uniform band (which is already the bandwidth optimum of the family), and no profile displaces the mode ratio *f*_2_/*f*_1_ below 5.77 (baseline 5.86 for the band, 5.51 and 6.09 for the through-cut and pocket), closing the mode-proximity route. The calculated width of 4.38 Hz is 60% higher than the mechanical limit 2*ζ f*_1_ = 2.73 Hz (matched-load model peak *f*_1_ = 68.2 Hz): the excess is electrical damping from the matched load. That is, the one broadening mechanism available in place, electromechanical coupling, is already fully deployed and best deployed by the uniform design (Figure 15). This ledger assumes topology-independent ζ; Section 5.7 quantifies how the conclusion moves if that assumption fails.

**Figure 16:**
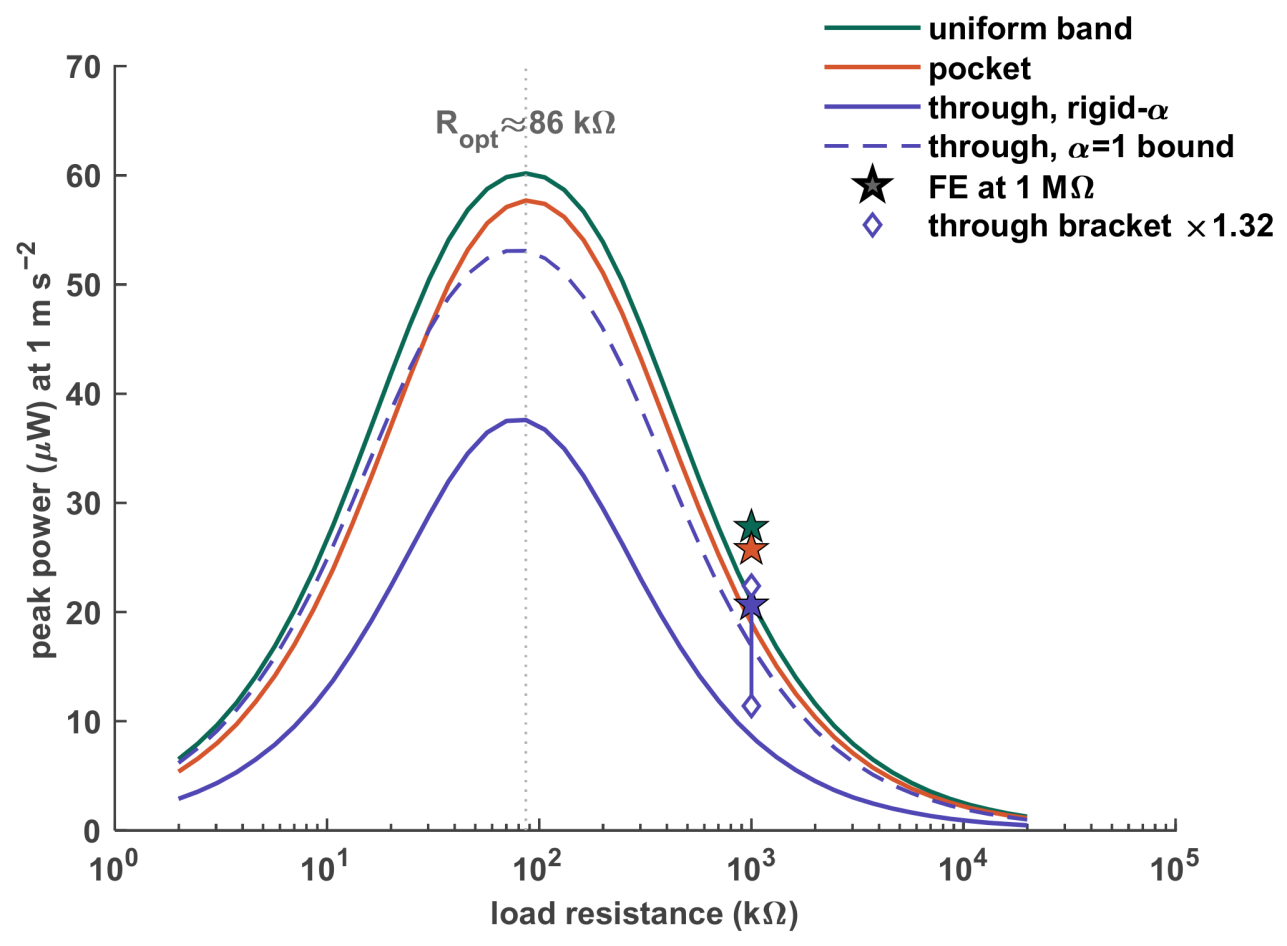
Model power versus load resistance for the three matched devices (the through-cut shown between its two bonding bounds); star mark: the finite element results at 1 MΩ and the open diamonds: the through-cut bracket carried into the finite element frame (×1.32, Section 4).

Load matching: Figure 16 sweeps the load for the three matched devices. All have a single optimum at *R_opt_* ≈ 86 kΩ ≈ 1/(*ω*_1_ *C_p_*), the impedance-match point, single-peaked because the devices are weakly coupled at ζ = 0.02. At this optimum, every design roughly triples its 1 MΩ output (band 60.2, pocket 57.7, through 37.6–53.1 µW across its bonding bounds), with the ordering unchanged. The load independence of the band’s dominance pre-empts the natural objection that the comparison was made at an arbitrary resistance. The curves have the usual weak-coupling shape and are worth presenting in full. The patch capacitance and the load form an RC divider. Power is maximal when the load impedance equals 1/*ω*_1_ *C_p_*. Since Cp belongs to the unchanged ceramic, the optimum hardly changes between designs. We see a single peak, not the split short-circuit/open-circuit pair that is seen in strongly coupled devices, which tells us that *k*^2^ (Equation (15)) is below 4*ζ* here. Running at 1 MΩ, well up the open-circuit branch, is what cost every design alike a factor of three in power.

### 5.6. Sensitivity of the Ordering: Damping, Coupling and Range

All three devices share the same assumed modal damping, *ζ* = 0.02 [25,26], but their roots differ in the mechanisms that set damping: open cells pump air, thin skins and slender ligaments concentrate cyclic strain, and the bonded areas differ by a factor of 2.6 in tie count; empirical work on architected cantilevers likewise identifies the damping loss factor as one of the discriminating contributions to output [46]. Resonant power falls with *ζ,* as 1/*ζ* at the matched load and more steeply on the open-circuit branch, so that the power margins are subject to inter-topology damping contrast. The exposure is quantifiable with the validated model. Keeping the pocket at *ζ* = 0.02 and inflating the damping of the band, the band–pocket power ordering reverses at a contrast of only 6% at 1 MΩ (3% at the matched load); the band–through ordering reverses at 13% (10%) under the force-continuity bound and at 62% (42%) under the rigid one. The exposure is one-sided. The ordering reserves only if the plain band is the more damped structure, whereas every mechanism above, cyclic strain concentrated in the pocket’s thin skin and slender ribs, air trapped in closed cavities, its larger bonded interface, acts to raise the pocket’s damping relative to the band’s. A 6% contrast in the band’s disfavor erases its load; the same contrast in the pocket’s disfavor raises the lead from 10.8% to 23.4% at 1 MΩ and from 4.3% to 12.9% at the same matched load (model). The physically expected direction of damping contrast, therefore, widens the margin rather than closing it; the power rankings are nevertheless to be read as equal-damping results, and damping identification belongs in the confirming experiment. The peak-stress ordering is far less exposed. For the common damping value, it is fixed by the mode shape and the sections, since every resonant amplitude scales by the same 1/ζ; under an inter-topology contrast, each device’s amplitude in Equation (14) scales as 1/ζ_i_ individually, and the ordering reserves only when the contrast reaches the stress margins itself: 24% (band-through, on peaks; the nominals), an order of magnitude beyond the 3–6% that reorder powers. The two conclusions are exposed in opposite directions; a more-damped pocket widens the band’s power lead while narrowing its stress margin, so the band dominates the pockets on both axes for any damping ratio ζ_pocket_/ζ_band_ between about 0.94 and 1.62.

We examined the extent of this ordering over design space by varying patch thickness and coverage at fixed matched geometries, with results collected in Table 6, evaluated at the finite element design vectors in Table 7. The order band pocket through-cut holds for *h_p_*/*h_s_* from 0.4 to 0.8 and patch coverage up to *L_p_* = 60 mm, all within the weak-coupling regime (*k*^2^/4*ζ* = 0.45–0.59). The two cellular designs converge to a near-tie only at the thinnest patch, and even then, the band still leads. 

The regime boundary is more important than any of these sweeps. Rerunning the matched trio with *h_p_* = 0.8 mm and *ζ* = 0.005, so that *k*^2^/4*ζ* reaches1.6–2.0, and letting each device operate at its own optimal load, the pocket overtakes the band, 192 against 184 µW, with the through-cut at 171. The mechanism is generic: once the coupling saturates, the resonant power is limited by forcing and mass, not the conversion efficiency, and the mass-preserving pocket root gains. The no-hole conclusion is, therefore, a weak coupling result, and we scope it as such. In strongly coupled load-optimized devices, the pocket family warrants fresh consideration.

The cantilever studied carries no tip mass, whereas most deployed harvesters do. The matching survives the addition unchanged and tightens: with a lumped tip mass added to the three matched substrates, their fundamentals agree to 0.05% at 5 g and 0.03% at 10 g against 0.12% without, because the tip mass takes over the modal inertia, and the treated zone’s share of it falls below 0.2%. The mechanisms that order the devices, the coupling arm, the active coverage and the neutral axis shift, are section properties that a tip mass does not alter. Running the matched trio with 5 g and 10 g tip masses in the segmented model leaves the power ordering band > pocket > through-cut unchanged at both loads (at 10 g and 1 MΩ), and lower k^2^/4ζ from 0.59 to 0.54, so the tip mass device sits deeper in the weak-coupling regime. The ordering, therefore, transfers, and the regime boundary of this section remains the quantity to check.

### 5.7. The Control Variable, Masses of the Devices, and an Alternative Frame

There are many controls, and matching the substrate-only fundamental is one. It is worth stating why it is the right control for this question. It keeps the transducer, the distal span and the clamp identical and equalizes exactly the quantity the root treatment is bought for, its compliance, to 0.3% (Section 2.3). The residual differences in device resonance and mass are then consequences of the treatment, part of what is being compared rather than a problem to be equalized away. The loop is closed by two complementary frames. First, mass is as follows: the devices weigh 15.4 (band), 15.0 (through) and 14.5 g (pocket) against 16.8 g solid, and the mass-normalized powers at 1 MΩ are 1.80, 1.38 and 1.77 µW/g against 1.02, so the ordering survives normalization, the lightest device, the pocket, nearly tying the band per gram. The canonical normalized power density *P*/*(Va*^2^*)* reads 1.26, 0.96 and 1.24 against the solid’s 0.71 µW mm^−3^ g^−2^, satisfying the reporting convention of [38,39]. Second, the designer’s frame of matched device resonance is as follows: retuning the cellular roots so that all three complete devices share the band’s fundamental (pocket *f* = 0.853, through-cut *f* = 0.878 in the model frame) gives 20.9, 18.0 and 19.2 µW for band, pocket and through-cut, so the band’s lead persists, by ≈9%, under the alternative control as well. Only the cellular pair’s internal order changes, consistent with their opposite coupling-versus-mass mechanics. Table 4 collects the three devices in every frame in which harvesters are conventionally compared. The band leads in all of them; the matched-compliance frame is not what produces the ordering but what allows it to be attributed.

### 5.8. Discussion

The design picture is summarized in three statements: The gain reported by the perforation is real. It is due to compliance. It is bought at a premium. It is real, since our through-cut beats the solid beam by 20%, where the literature places such devices; it is borrowed, because the active ingredient is compliance under the electrode. And compliance does not care how it is manufactured. It is expensive because, against the cheapest way of obtaining the same compliance, every cellular elaboration we could build, holes of any footprint or orientation, pockets of any depth, stepped or graded profiles, returned lower power, higher stress, or both. We would go further: the solid-beam baseline that pervades the field has been quietly overstating what architected substrates contribute, and that the matched-compliance thinned root should be the standard control for such claims. This study gives the protocol and the numbers to implement it. The envelope of that recommendation is made explicit rather than implicit: it is valid across the tested patch thicknesses and coverages in the weak-coupling regime, it is conditional on inter-topology damping contrasts being below the few percent thresholds of Section 5.6, and it is reversed in favor of the pocket in the strongly coupled load-optimized regime.

Each penalty has a mechanism written down in Section 2.1 and Section 5.3: the through-cut pays in its ligaments, where the net section magnifies stress by *b*/*b_n_* and at its electrode, a quarter of which is bonded to nothing; the pocket pays through its shifted neutral axis, which shortens the coupling lever arm. The second penalty proved smaller than the textbook assumption implies, and the finite element lands near the optimistic bound (inside the offset-corrected bracket, at an effective coverage of about 0.9). So, ceramic spanning a hole still transmits most of its membrane strain. If you model perforated harvesters under an inactive-electrode assumption, you are over-penalizing them. This is a useful correction, even if you keep your holes. Closed skins rebound and then mechanically undo the good work, moving the neutral axis toward the ceramic so that the critical fiber works harder and the transducer sees less. The band pays no penalty. Another comment from the validation is worth passing along: solid side margins on a thinned band are fully effective for global stiffness but are avoided by the local plate stress, two regimes we could bracket to within a percent and a caution for anyone homogenizing narrow architected strips.

The optimizations are essentially one sentence: the results support fixed compliance and fixed transducer; root geometry tunes the frequency and sets the stress and does essentially nothing else. Power is shifted by roughly the model’s own resolution across the entire root design space. Bandwidth is unchanged because the one broadening mechanism available (electrical damping through the coupling) is already used up fully by the uniform design. There will have to be other levers altogether that provide real power or bandwidth gains, and none of the usual suspects are on board with perforation either.

The limitations are the usual ones and one special one. The features are square cornered and the screening meshes deliberately economical; the orderings are mesh stable, and we have said where the corner effects enter, but a manufactured device wants filleted geometry and design-grade stress analysis. The conformal tie is an idealized zero-thickness bond. A finite-compliance adhesive layer would soften the force-continuity bound (bonding stiffness is modeled explicitly in the perforated-chirality literature [15]), and one adhesive layer case belongs to the experimental follow-up. The through-cut exhibits laminate behavior, which is bracketed rather than pinned, and we consider this as much a finding as a gap. A bench test adds exactly the two scalars the sensitivity study isolates, the per-device damping contrast and the effective coverage *α* of an electrode bridging machined holes; both enter the validated chain as measurable inputs, so a modal test and one loaded frequency response convert directly into a recalibrated comparison, sharpening the ordering rather than re-deriving it. That experiment is unusually cheap because the band plays just one milling pass. Running it is the obvious next step.

## 6. Conclusions

This study asked what a hole pattern contributes to piezoelectric cantilever harvesters beyond the compliance its adds and answered it with control the literature lacks: every perforated root was benchmarked against a uniformly thinned root of identical substrate compliance. Under that control, in the weak-coupling regime of the devices studied (k^2^/4ζ = 0.45–0.59), the uniformly thinned band matched or outperformed every perforated root. It delivered 62% more power than the solid reference at equal footprint (27.8 vs. 17.2 µW at 1 MΩ, FE), 8% more than the closed-skin pocket and 34% more than the through-cut, at the lowest peak substrate stress of the three treated roots, with 77% higher normalized power density and 76% more power per gram than the solid beam. It led in all five frames in which harvesters are conventionally compared. The optimizations of footprint, orientation, depth, thickness profile and load ended at the no-hole boundary in both the matched and frequency-free frame, with five random restarts and an independent global search agreeing. Three-dimensional coupled finite element analysis confirmed the model at nine anchors, including three optimized deigns: the best real-hole design-matched compliance delivered 16% less power than the plane band, and no hole design entered the power stress frontier. The results reduce to three section-level mechanisms that a designer can act on. Thinning under the electrode lengthens the coupling lever arm and concentrates curvature where the transducer is, and through-cut pays twice, in ligaments whose net section magnifies stress and at an electrode, a quarter of which is bonded to nothing; pockets shifts the neutral axis toward the ceramic and shorten the lever arm. The bonding of ceramic across a hole was bracketed between rigid and force-continuity idealization, and the finite element landed near the force-continuity bound, at an effective active coverage of about 0.9. The design guidance that follows is concrete. (1) Default: thin the root uniformly under the electrode; the matched design removes 37% of the substrate thickness over the treated zone and is the lowest-stress member of the family. (2) Power priority: front-load the depth. A four-step profile (0.66, 0.66, 0.53 and 0.22 of the thickness removed, clamp side first) gives 9% more power than the uniform band at matched load (FE) for 1.6-times its peak stress; choose it where the fatigue margin allows and note that both options are thinned roots. (3) Do not perforate a weakly coupled root: the best hole design available at matched compliance costs 16% of the power (FE); for the pocket, there is more peak stress. (4) The exception is strong coupling: with a thick patch, low damping and the load optimized (k^2^/4ζ ≈ 1.6–2.0), the mass-preserving pocket overtakes the band (192 vs. 184 µW), so the coupling regime should be checked before a cellular root is dismissed. (5) Operate at the matched load, not at nominal 1 MΩ (FE), and the beam model, which is 32% below the finite element on the open-circuit branch, is within 7% of it at the matched load, so it is adequate for the load-optimized design. (6) The conclusions transfer to tip-mass cantilevers: the matching tightens to 0.03$ at 10 g, the ordering persists, and k^2^/4ζ falls slightly, keeping the device in the weak-coupling regime, so the recommendation holds with or without tip mass. (7) Power ordering is an equal-damping result that reverses if the band is about 6% more damped than the pocket; every damping mechanism identified acts in the opposite direction, and stress ordering survives contrasts one order of magnitude larger; the recommendation, therefore, stands at equal or band-favorable damping. (8) The half-power width of the matched-load band is 4.38 Hz, 1.6-times its mechanical floor of 2ζf_1_ = 2.7 Hz, because the matched load injects electrical damping. For practice, the implications are direct. A self-powered sensor node built on this class of harvester gains 62% in its power budget from a plain milled recess rather than from a lattice of holes, with lower stress and cheaper process, and a designer who wants the last 9% can profile the recess without changing the tooling. The section mechanics do not depend on scale, so the rule applies to micromachined harvesters as much to the macro-scale devices analyzed here, where etching a lattice is a more expensive route and thinning is less. The matched-compliance protocol itself is offered as the control that future claims for the cellular, auxetic or kirigami roots should pass: any gain that survives comparison with an equally compliant thinned root is attributable to the pattern; any that does not is compliance under another name.

The limits are stated. The results are computational, obtained at an assumed damping ratio common to all devices, in the weak-coupling regime and on square-cornered features without fillets; a tip mass enters only through the check in Section 5.6. No experiment was performed. Future work will address the two quantities the model cannot supply. A modal test and loaded frequency response on the machined band and pocket specimens will identify the damping contrast and the effective coverage directly and thereby confirm or recalibrate the ordering reported here; the strongly coupled, load optimization prototype will then extend the comparison into a regime, where the pocket family deserves fresh consideration.

## Figures and Tables

**Table 1:** Summary of the three-dimensional finite element model.

Item	Setting
Substrate element	3D structural quadratic tetrahedron
Piezoceramic element	3D coupled-field quadratic tetrahedron (UX, UY, UZ, VOLT)
Patch bonding	interface constraint equations (conformal; glued cross-check 0.01%)
Load element	circuit resistor across equipotential electrodes
Substrate material	isotropic, *E_s_* = 100 GPa, ν = 0.3, ρ*_s_* = 7165 kg m^−3^
Piezoceramic	PZT-5A2 anisotropic *c^E^*, *e*, ε*^S^*; poled through thickness
Modal analysis	Block–Lanczos
Harmonic analysis	full; ζ = 0.02 at resonance; two-pass (FRF, then peak field)
Boundary/load	clamped at *x* = 0; lower electrode grounded; 1 m s^−2^; *R* = 1 MΩ
Typical model size	5.3 × 10^4^ nodes, 2.6 × 10^4^ quadratic tetrahedra (untreated device)
Self-checks	volume (<0.2%), centroid, tie count, patch-engagement gate

**Table 2:** Coupled-field results at matched substrate compliance (finite element; 1 m s^−2^, 1 MΩ).

	Solid (Ref.)	Thinned Band	Through-Cut	Closed-Skin Pocket
device *f*_1_ (Hz)	86.9	70.85	75.72	67.46
power (µW)	17.19	27.79	20.67	25.76
substrate stress (MPa)	2.63	4.60	5.70	7.45
piezo stress (MPa)	2.12	2.95	2.88	3.45
device mass (g)	16.8	15.4	15.0	14.5
power per mass (µW g^−1^)	1.02	1.80	1.38	1.77
NPD (µW mm^−3^ g^−2^)	0.71	1.26	0.96	1.24
tie equations (–)	23,220	23,220	16,863	44,340

All four columns are calculated in the same coupled finite element pipeline. Solid column substrate stress is a clamped-edge peak (beam-theory nominal ≈ 1.3 MPa, corner factor ≈ 2). Treated-device values are first-feature-row peaks (Section 5.3).

**Table 3:** Model-versus-finite-element comparison by design at 1 MΩ (frequencies in Hz, powers in µW; κ = PFE/Pmodel; stresses from the substrate-only screen where available).

Design	f1 Model/FE (Hz)	Δf	*p* Model/FE (µW)	κ	σ Closed-Form/FE (MPa)
solid	83.5/86.9	−3.9%	13.2/17.19	1.31	1.3 nominal/2.63 clamp-edge (Kt ≈ 2)
band β = 0.375	67.9/70.9	−4.2%	21.1/27.79	1.32	4.57/4.61 (+0.9%)
pocket f = 0.909, β = 0.75	65.7/67.5	−2.7%	19.0/25.76	1.36	5.3 nominal/6.4 (−17%, corner)
through f = 0.70	74.7/75.7	−1.3%	8.6–16.9 (bracket)/20.67	1.32 applied → 11.4–22.4	4.3 nominal/5.40 (−20%, corner)
deep band β = 0.557 (frequency-free optimum)	63.2/65.3	−3.2%	22.6/30.68	1.36	—
best real holes f = 0.893, β = 0.892 (matched)	69.0/67.9	+1.6%	18.2/23.22	1.28	—
max-power profile (matched)	63.4/65.1	−2.6%	22.1/30.06	1.36	—
band at 86 kΩ (matched load)	—	—	60.2/64.39	1.07	—
profile at 86 kΩ (matched load)	—	—	63.3/70.29	1.11	—

**Table 4:** Power ordering across comparison frames (band lead over pocket/over through-cut).

Comparison Frame	Band	Pocket	Through-Cut	Band’s Lead
matched substrate compliance, 1 MΩ (FE, Table 2)	27.8 µW	25.8	20.7	+8%/+34%
matched device frequency, 1 MΩ (model)	20.9	18.0	19.2	+16%/+9%
optimal load, frequency free (model)	60.2	57.7	37.6–53.1	+4%/+13–60%
mass-normalized, µW g^−1^ (FE)	1.80	1.77	1.38	+2%/+30%
normalized power density, µW mm^−3^ g^−2^ (FE)	1.26	1.24	0.96	+2%/+31%

**Table 5:** Optimization settings for every search in this study.

Search	Variables	Bounds	Constraints	Algorithm/Start	Stopping/Evaluations
frequency-free holes (Section 5.2)	fx, fz, β	0.15 ≤ fx, fz ≤ 0.999; 0.05 ≤ β ≤ 0.999	skin (1−β)h_s ≥ 0.10 mm (β ≤ 0.90); ligament (1 − fz)pz ≥ 0.5 mm (fz ≤ 0.9375)	Nelder–Mead, box-clipped, from (0.6, 0.6, 0.5); DE cross-check	x_tol = f_tol = 5 × 10^−3^, ≤60 iterations; ≈100 evaluations (DE: 624)
inner peak search	excitation frequency	[f1 − 9, f1 + 3] Hz	—	bounded scalar minimization of −|V|^2^/2R	10^−3^ Hz
sweeps and map (Section 5.2)	fx (8 pts), fz (12 pts) on [0.15, 0.98]; map 10 × 10 on fz ∈ [0.15, 0.98], β ∈ [0.10, 0.95]	—	—	grid	—
matched landscape (Section 5.5)	fx, fz; β solved per point	9 × 9 grid on [0.15, 1.0]	f1,sub = 44.885 Hz by bracketed root finding in β; load optimized per point	grid + Nelder–Mead refinement; 5 random restarts; DE cross-check	5 × 10^−3^; restarts agree to β = 0.556 ± 0.006
thickness profile (Section 5.5)	four cell thicknesses	0.10 mm ≤ h_r, k ≤ h_s	f1,sub = 44.885 Hz (equality)	equality-constrained gradient search from the uniform band	5 × 10^−3^
stress evaluation	—	13 stations across the treated zone; maximum reported	—	—	—

**Table 6:** Generalization sweep: model power (µW) at 1 MΩ (last row at per-device Ropt) for finite element design vectors; through-cut under the force-continuity bound.

Configuration	*k*^2^/4ζ	Band	Pocket	Through	Order
*h_p_* = 0.2 mm	0.45	13.5	11.7	11.7	band first, pair converges
*h_p_* = 0.4 mm (base)	0.59	21.1	19.0	16.9	band > pocket > through
*h_p_* = 0.6 mm	0.57	20.4	18.9	16.4	band > pocket > through
*h_p_* = 0.8 mm	0.50	17.3	16.3	14.1	band > pocket > through
*L_p_* = 60 mm	0.58	16.5	15.0	13.7	band > pocket > through
*h_p_* = 0.8, ζ = 0.005, *R_opt_*	1.6–2.0	184	192	171	pocket overtakes

**Table 7:** Matched design vectors (substrate only f1 = 44.885 Hz).

Topology	*f_x_*	*f_z_*	β	Remaining Material
thinned band	–	–	0.375	*h_r_* = 0.625 mm
through-cut	0.70	0.70	1	ligaments 2.4 mm
pocket	0.909	0.909	0.75	skin 0.25 mm

## Data Availability

Data are archived at Zenodo (DOI 10.5281/zenodo.22816626, https://doi.org/10.5281/zenodo.22816626) with restricted access and are available from the corresponding author upon reasonable request.

## Nomenclature

*L*, *b*, *h_s_*	beam length, width, substrate thickness	*f_x_*, *f_z_*	hole footprint fractions
*h_p_*, *L_p_*	patch thickness, patch length	Β	feature depth fraction
*x_m_*, *z_m_*	array margins	*h_r_*	remaining band thickness (1 − β)*h_s_*
*p_x_*, *p_z_*; *N_x_*, *N_z_*	cell pitches; counts	*b_n_*	net section width
*f*_1_, ζ	fundamental; damping ratio	A	active electrode coverage
*R*, *C_p_*	load; patch capacitance	θ*_r_*	modal coupling
${\bar{E}}_{p}$, ${\bar{e}}_{31}$, ε*^S^*	reduced patch constants	σ_0_	solid-beam reference stress
